# Uniaxial mechanical stretch properties correlated with three-dimensional microstructure of human dermal skin

**DOI:** 10.1007/s10237-023-01813-3

**Published:** 2024-02-07

**Authors:** Mengyao Zhou, Patrick José González, Ludo Van Haasterecht, Alperen Soylu, Maria Mihailovski, Paul Van Zuijlen, Marie Louise Groot

**Affiliations:** 1 Faculty of Science, Department of Physics, Laserlab, Vrije Universiteit Amsterdam, De Boelelaan 1105, 1081HV Amsterdam, The Netherlands; 2 Burn Center and Department of Plastic, Reconstructive and Hand Surgery, Red Cross Hospital, Mozartstraat 201, 1962 AB Beverwijk, The Netherlands; 3 Department of Plastic, Reconstructive and Hand Surgery, Amsterdam University Medical Center (UMC), Location Vrije Universiteit Amsterdam, De Boelelaan 1117, 1081 HV Amsterdam, The Netherlands; 4 Pediatric Surgical Centre, Emma Children’s Hospital, Amsterdam University Medical Center (UMC), Location University of Amsterdam, Meibergdreef 9, Amsterdam, The Netherlands; 5 Amsterdam Movement Sciences (AMS) Institute, Amsterdam University Medical Center (UMC), Location Vrije Universiteit Amsterdam, Meibergdreef 9, Amsterdam, The Netherlands

**Keywords:** Human skin, Second harmonic generation, Uniaxial skin stretch, Collagen fibers, Elastin fibers, Mechanical properties

## Abstract

The intact and healthy skin forms a barrier to the outside world and protects the body from mechanical impact. The skin is a complex structure with unique mechano-elastic properties. To better direct the design of biomimetic materials and induce skin regeneration in wounds with optimal outcome, more insight is required in how the mechano-elastic properties emerge from the skin’s main constituents, collagen and elastin fibers. Here, we employed two-photon excited autofluorescence and second harmonic generation microscopy to characterize collagen and elastin fibers in 3D in 24 human dermis skin samples. Through uniaxial stretching experiments, we derive uni-directional mechanical properties from resultant stress-strain curves, including the initial Young’s modulus, elastic Young’s modulus, maximal stress, and maximal and mid-strain values. The stress-strain curves show a large variation, with an average Young’s modules in the toe and linear regions of 0.1 MPa and 21 MPa. We performed a comprehensive analysis of the correlation between the key mechanical properties with age and with microstructural parameters, e.g., fiber density, thickness, and orientation. Age was found to correlate negatively with Young’s modulus and collagen density. Moreover, real-time monitoring during uniaxial stretching allowed us to observe changes in collagen and elastin alignment. Elastin fibers aligned significantly in both the heel and linear regions, and the collagen bundles engaged and oriented mainly in the linear region. This research advances our understanding of skin biomechanics and yields input for future first principles full modeling of skin tissue.

**Supplementary Information:**

The online version contains supplementary material available at 10.1007/s10237-023-01813-3.

## Introduction

Skin is the largest organ of the human body and consists of the epidermis and dermis. The dermis is a flexible layer that prevents the epidermis from rupturing or tearing by resisting pressure and stretching (Yang et al. 2015). The dermis provides tensile strength and elasticity to the skin through an extracellular matrix composed of collagen and elastin fibers, embedded in hyaluronan and proteoglycans (Elsner et al. 2001; Wilkes et al. 1973; Dwivedi et al. 2022; Joodaki and Panzer 2018). Collagen type I is the major dermal constituent and contributes to around 60–80% of the fat-free dry mass and 18–30% of the volume of dermis (Ebling 1992; Reihsner et al. 1995). It is composed of triple, left-handed, helices of polypeptide strands, forming a right-handed helix. Collagen molecules self-assemble and crosslink into fibrils with typical lengths of 1 $\upmu$m, which in turn associate into fibers of 10 $\upmu$m length and further into bundles of fibers that provide the tissue with its tensile properties (Xu et al. 2008). Elastin fibers comprise 4% of the fat-free dry mass and 1% of the volume of the dermis (Ebling 1992; Hult and Goltz 1965) and contribute to the tensile properties of skin (Sarah and Régis 2012).

Understanding the complex mechanical behavior of skin is important for many clinical applications (e.g., scar management) (Hendriks 2001; Hochberg et al. 2009; Blair et al. 2020) and the development of biomimetic materials (Shi et al. 2023; Veera Krishna et al. 2020; Zhu et al. 2020). The typical J-shaped stress–strain curve has been demonstrated through a range of experimental techniques, including uni-, bi-, and multi-axial stretching (Kumaraswamy et al. 2017; Dwivedi et al. 2020a, b; Wan 1994; Jun et al. 2021; Sun et al. 2018; Kvistedal and Nielsen 2009), suction (Diridollou et al. 2000) and bulging (Lakhani et al. 2020; Tonge et al. 2013). This curve arises from the synergistic interplay of the two main structural proteins: collagen and elastin (Jansen et al. 2018; Chow et al. 2014). Although direct structural connections between elastin and collagen fibers have not been observed, collagen fibers appear to wind around elastin cores (Xu et al. 2008). As isolated collagen fibers are nearly three orders of magnitude stiffer than elastic fibers (Chow et al. 2014), it is normally considered that elastin fibers play a leading role in withstanding deformation of skin at lower stress, while collagen fibers become the major load bearing component at higher stress (Daly 1982). The J-shaped behavior is also shown in studies that focus on the mechanical behavior of isolated collagen fibrils (Svensson et al. 2010; Yang et al. 2022; Fratzl et al. 1998). The strain within these fibrils was found to be considerably smaller than in the whole tendon. This phenomenon is still understood poorly, but it points toward the existence of additional gliding processes occurring at the interfibrillar level (Fratzl et al. 1998).

In murine skin tissue, Lynch’s group (Lynch et al. 2017a, b; Bancelin et al. 2015; Allain et al. 2019) observed a J-shaped stress–strain curve with collagen alignment occurring continuously during stretching. From these studies the view emerges that stretching of skin is enabled by orientation of collagen fibers, and the smooth nonlinear response, as opposed to abrupt transition from elastic to stiff material is due to gradual recruitment of fibers, possibly due to structural effects of the fibers network in interaction with the surrounding nonlinear matrix. These findings were recently corroborated by the first measurements on fresh unprepared human skin by our group (van Haasterecht et al. 2023), using second harmonic generation (SHG) microscopy. The results showed a wide variability of both measured stress–strain curves and collagen alignment. Also, mean orientation indices at the different stages of the stress–strain curves (toe, heel, and linear) showed a significant increase in collagen alignment during the linear part of the mechanical response.

**Fig. 1:**
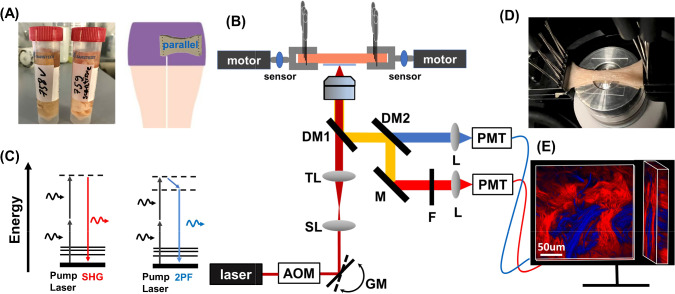
**A** Human skin samples from cadaver thighs and cutting orientation. **B** Schematic diagram of the experimental setup: The laser has a central wavelength of 1050 nm with pulse duration less than 80 fs; *AOM* acousto-optic modulator, *GM* galvo mirrors;* SL* scan lens, *TL* tube lens with adjustable focus, DM1 dichroic mirror reflecting backscattering signals from fundamental photons, DM2 dichroic mirror splitting TPEF and SHG channels, *M* mirror, *F* bandpass filter, F520/35, *L* focusing lens, *PMT* photomultiplier tube detectors. **C** Energy level diagram of the SHG and TPEF process. **D** Image shows the deformation of skin sample at 115% stretching. **E** 3D volume image of collagen and elastin fibers from human thigh skin. Red color represents collagen fibers and blue color represents elastin fibers

In this study, we aim to exploit the variety in mechanical and microstructural properties of human skin of different subjects, to learn more about the determining factors for human skin mechanical behavior. We extended the number of human thigh dermis samples to 24 to obtain insight into the variation in observations and the underlying structural causes. To capture a comprehensive view, we integrated two-photon excited autofluorescence (TPEF) imaging with our fast SHG imaging setup during uniaxial stretching. This allowed us to concurrently monitor the reactions of collagen and elastin fibers.

## Materials and methods

### Human skin tissue processing

All procedures on human tissue were performed with the approval of the Medical Ethical Committee of the Amsterdam UMC (Biobank reference number 2017.098). All samples came from people who agreed to donate their bodies for scientific research after they died. Skin tissue samples were cut from the upper thigh of the cadavers. After resection, the samples were placed in a tube with culture medium: 98% of Gibco Roswell Park Memorial Institute (RPMI) 1640 Medium (Thermo Fisher Scientific) and 2% of penicillin-streptomycin (10,000 U/mL), as shown in Fig. 1A, and transported to the laboratory for the experiments.

The sample was cut parallel to the thigh circumference direction, as illustrated in Fig. 1A, and formed into a standard tensile test specimen, also called T-bone or dog-bone shape. This shape ensures homogeneous uniaxial tensile load in the center of the test specimen (Ducourthial et al. 2019; Bancelin et al. 2015). The prepared specimen had dimensions of approximately 40 mm in length, 20 mm in width, and 0.8 mm in thickness. The split skin graft is composed of the epidermis and the reticular dermal layer, which allows for targeted analysis of the reticular dermal layer at specific depths. This procedure guarantees consistent sample thickness, consequently creating uniform in-plane tension. The sample was secured to a custom-built stretching device using 12 steel needles and covered with a 0.17-mm-thick glass coverslip to maintain a flat sample surface during SHG/TPEF imaging (Fig. 1B). The reticular dermis layer of the sample was positioned facing the objective lens.

### SHG/TPEF imaging and mechanical loading

The experimental setup consisted of a laser scanning nonlinear microscope and a custom-built stretching device, which is illustrated in Fig. 1B. The laser source (during the experiment three different laser sources were used: FSP-03, Seed Lasers; BIOLIT 2, Litillit; Tidal, Valo Innovations) produced femtosecond pulses with pulse duration less than 85/80/50 fs, respectively, and a central wavelength of 1050 nm. An acousto-optic modulator (AOM, MT250-A0.5-1064, Opto Electronic) was used as pulse picker to reduce the repetition rate of the laser from 10–15 to 1 MHz, in order to keep the average power low, but the peak power high enough to excite nonlinear signals. A pair of galvo mirrors (GM) was used to scan the laser in *x-* and *y*-direction to generate 2D images. 3D information of the sample was obtained with an electrically focus tunable lens (TL, EL-10–30-Ci-NIR-LD-MV, Optotune Switzerland AG), which enables to rapidly adjust the focal length from 0 to 30 $\upmu$m. The beam was focused on the sample by a $40\times /1.30$ (Nikon S Fluor, Nikon) oil-immersion objective, resulting in a focus of size 0.4 $\upmu$m $\times \,0.4 \,\upmu$m $\times 2\,\upmu$m. The generated signals were detected in epi-direction, filtered from the 1050 nm fundamental photons by a dichroic mirror (DM1, FF872-Di01, Semrock), and subsequently divided into two channels through another dichroic mirror (DM2, LP580, Semrock). A bandpass filter (F, FF01-520/35-25, Semrock) was placed in the detection arm of the SHG. Two photomultiplier tubes (PMT, TPEF: H10721-20, SHG: H16201-40; Hamamatsu, Japan) were used to detect the signals. Microscopy data were recorded using in-house developed LabView software. In the SHG process, signals result from the conversion of an incident photon pair into one photon with twice the energy and half the wavelength (Kuzmin et al. 2016), as shown in Fig. 1C. Collagen fiber is an efficient generator of SHG signal because of its non-centrosymmetric structure whereas elastin shows autofluorescence that we excited via two-photon absorption. Figure 1E shows a 3D volume representation image of collagen and elastin fibers from human thigh skin, which was processed by the 3D script plugin of ImageJ, displaying SHG signals in red, and TPEF signals in blue.

The stretching device was placed over the objective on a 3D moving stage as shown in Fig. 1B. The skin samples were stretched up to 125–150% of their initial length (depending on the properties of the samples) at a speed of 0.5 mm/s by two motors, while the force and the motor displacement were continuously measured by two sensors. The strain $\varepsilon$ is a measure of how much a material deforms when subjected to an external force and is given by $\varepsilon$ = $\Delta L/L_0$, where $L_0$ is the initial length of the sample and $\Delta L$ is the change in length. The nominal stress $\sigma$ is a measure of how much force is applied to a material per unit area and is given by $\sigma$= $F/S_0$ = $F/(w_0 \times e_0)$, where *F* is the measured force, $S_0$ is the initial cross section and $w_0, e_0$ are the initial width (20 mm) and thickness (0.8 mm) of the sample (Lynch et al. 2017b; Wahlsten et al. 2023; Jun et al. 2021). The experiments were conducted in an enclosure of the microscope where the ambient temperature and relative humidity were kept at 23^∘^C and 44%. The humidity of the sample remained constant throughout the experiment as the duration was kept short and the protective objective cap and coverslip were in contact with the dermal side of the sample, thereby reducing water evaporation.

**Fig. 2:**
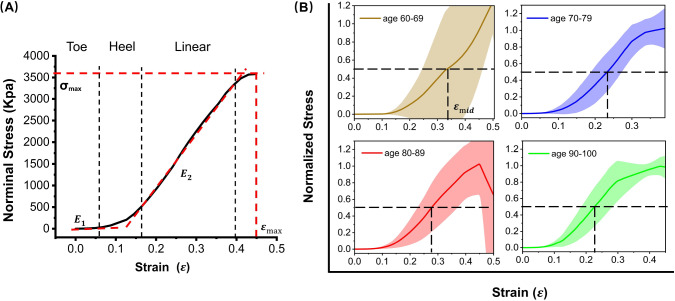
Uniaxial stretching data for human thigh dermis skin. **A** A representative stress–strain curve of human dermis skin (S5), where $E_1$ is the initial Young’s modulus, the slope of the curve in the toe region, $E_2$ is the elastic modulus (Young’s modulus), the slope of the linear region of the curve, and $\sigma _\mathrm{{{max}}}$ and $\varepsilon _\mathrm{{{max}}}$ are maximum stress and maximum strain before failure. **B** Average normalized stress–strain curves for different age groups of donors, with shaded areas representing standard deviations. The dashed lines illustrate 50% of the maximum stress, corresponding to the strain denoted as $\varepsilon _\mathrm{{{mid}}}$. The individual stress–strain curves of each group can be found in SI (Figure S1)

Two loading tests were performed for each sample: one involving continuous stretching to obtain the smooth stress–strain curves, as shown in Fig. 2, and another involving stepwise stretching during loading for imaging, as shown in Fig. 6D. In the latter, we used a step size of 2 mm. After each step, we shortly paused the motors (5 – 10 min) for multiphoton imaging. The imaging depth ranged up to 30 $\upmu$m with 1 $\upmu$m sampling intervals. Considering the differing refractive indexes between the objective immersion oil ($n_1 = 1.518$) and skin sample ($n_2 = 1.37$), the actual depth was found to be half of the apparent depth (Visser et al. 1992). Therefore, the actual axial sampling resolution was determined to be 0.5 $\upmu$m/pixel. The field of view (FOV) was 500 $\times$ 500 $\upmu$m^2^, and the lateral sampling resolution was 0.5 $\upmu$m/pixel. For an accurate data processing, we clipped off the dark invalid areas around the edges from 500 $\times$ 500 $\upmu$m^2^ raw data to 400 $\times$ 400 $\upmu$m^2^. Figure 1D shows the deformation of a sample at 15% strain where the sample’s width is reduced, and the middle part of the sample was slightly raised. We gently pressed the sample on the coverslip after each stretching step to match the imaging depth, but it was difficult to image the same region of interest (ROI) of the sample in 3D.

### Quantitative analysis of collagen and elastin fibers alignment

In order to get insight into the reorganization of collagen and elastin fibers during stretching, we calculated the 3D orientation of collagen and elastin fibers pixel by pixel using a weighted vector summation algorithm (Liu et al. 2015, 2017). Let $\theta$ and $\varphi$ be the azimuthal and polar angles used to define a 3D orientation (See Figure S5 in supplementary information (SI)). The azimuthal angle $\theta$ is determined by projecting fibers to the *xy* plane. The polar angles $\varphi$ are related to $\beta$ and $\gamma$, which are the azimuthal angle in the *xz* and *yz* planes, respectively, and can be calculated by the following formula (Liu et al. 2017): $\tan ^2 \varphi =1/(\tan ^2 \beta ) +1/(\tan ^2\gamma).$

To determine the $\theta$, $\beta$ and $\gamma$ angles, a 11 $\times$ 11 $\times$ 11 voxel window was selected, which was first projected onto the *xy*, *xz* and *yz* planes, respectively, and then all vectors passing through the center pixel of the window size are weighted by their length and intensity fluctuation along their direction. The software used a sampling ratio parameter of 1 between the *xy* and *z* dimensions to match both lateral and actual axial resolutions of 0.5$\upmu$m/pixel. Based on directional statistics (Liu et al. 2017; Mardia et al. 2000), the center of mass for axial data (orientation from 0 to $180^{\circ }$ ) is $(\bar{C},\bar{S},\bar{Z})$, where

1 $$\begin{aligned}&\bar{C}=(1/n)\sum _{j=1}^{n} \left( b_j/\sqrt{(1+b_j^2 )}\cos (2\theta _j)\right) , \end{aligned}$$

2 $$\begin{aligned}&\bar{S}=(1/n)\sum _{j=1}^{n} \left( b_j/\sqrt{(1+b_j^2 )}\sin (2\theta _j)\right) , \end{aligned}$$

3 $$\begin{aligned}&\bar{Z}=(1/n)\sum _{j=1}^{n}SI/\sqrt{1+b_j^2}, \end{aligned}$$

4 $$\begin{aligned}&b_j = \sqrt{1/\tan ^2 \beta _j +1/\tan ^2 \gamma _j}, \end{aligned}$$

5 $$\begin{aligned}&SI = {\left\{ \begin{array}{ll} -(\varphi -90)/\left| \varphi -90 \right| &{} \varphi \ne 90^{\circ },\\ 1 &{} \varphi = 90^{\circ }. \end{array}\right. } \end{aligned}$$

In the above, the summations extend over all pixels in a *z*-stack, as we want to capture the overall orientation of fibers. The mean resultant length, which is defined as the three-dimensional orientation index (3DOI), $\bar{r}_{3D}=\sqrt{\bar{C}^2 +\bar{S}^2+\bar{Z}^2}$, indicates the overall fiber alignment. The range from 0 to 1 corresponds to fibers ranging from strongly disordered to highly aligned, respectively.

The representative fiber angular distribution of $\theta$ is obtained by summing and normalizing all layers within a *z*-stack. Following this, the representative distribution is modeled using a mixture of two von Mises distribution, which is defined as Sadeghinia et al. (2023):

6 $$\begin{aligned} \rho _{vm}&= b+w\frac{1}{\pi } {\rm{exp}}\{a_1 {\rm{cos}}(2(\theta -\alpha _1))\}/I_0(a_1) \nonumber \\&\quad +(1-w)\frac{1}{\pi } {\rm{exp}}\{a_2 {\rm{cos}}(2(\theta -\alpha _2))\}/I_0(a_2), \end{aligned}$$

where *b* is a constant, $w\in [0,1]$ is the weight factor, $\alpha$ and *a* are the mean fiber angle and concentration parameter, respectively, and the subscript indicates the first or second fiber family and $I_0$ is first kind modified Bessel function of zero order.

### Structural measurements of collagen and elastin fibers

To enhance image contrast, we used limited adaptive histogram equalization (CLAHE) as implemented in MatLab. Fiber bundle thicknesses were measured manually using ImageJ. Five random ROIs were selected per image, with four images measured per stack. The average fiber thickness of each sample was obtained by averaging these 20 data points. For the fiber density, we first obtained a binary mask that selected all fiber regions by setting the threshold to 0.45 times of the mean value of the intensity of each *z*-stack. Then, the pixels number of the fibers was taken to be equal to the sum of the matrix of the binary mask, while the total number of pixels in the entire *z*-stack was 1000$\times$1000$\times$30. Subsequently, the density of fibers was calculated by dividing the number of fiber pixels by the overall number of pixels in each *z*-stack.

### Statistical analysis

Statistical analysis was performed using Origin (Version OriginPro 2022b, Academic.). Groups were compared using analysis of variance (ANOVA) followed by seven kinds of methods, including Tukey, Bonferroni, Dunn-Sidak, etc. The significance threshold was set at $P < 0.05$ and trend threshold at $P < 0.01$.

## Results

### Stress–strain curves

We conducted stress–strain measurements on 24 human dermis skin thigh samples. In order to prevent sample rupture, samples were stretched to a maximum strain of 25–50% (depending on the properties of the samples). Figure 2A shows the stress–strain curve of sample S5, exhibiting a classic J-shaped curve consisting of three regions as indicated by the black dashed lines: toe, heel and linear region typical for collagen (Fratzl et al. 1998; Gutsmann et al. 2004). In the toe region, the stress is minimal, and the stress–strain relationship obeys Hooke’s law with initial Young’s modulus of $E_1$. The existence of this “minimal-stress” region is why the curve is called J-shaped (Mitsuhashi et al. 2018). In the linear region, the stress linearly increases with strain, with elastic Young’s modulus $E_2$. The region where the two lines smoothly connect is called the heel. Figure 2B displays the average normalized stress–strain curves for different age groups of donors, with shaded areas representing standard deviations. The dashed lines illustrate 50% of the maximum stress, corresponding to the strain denoted as $\varepsilon _\mathrm{{{mid}}}$. All stress–strain curves of each group can be found in SI (Figure S1).

In order to quantitatively compare the mechanical properties, five key characteristics from the stress–strain curves were identified (illustrated in Fig. 2A, B): the initial Young’s modulus ($E_1$, or the slope of the curve in the toe region); the elastic Young’s modulus ($E_2$, or the slope of the linear region of the curve); maximum stress ($\sigma _\mathrm{{{max}}}$); maximum strain ($\varepsilon _\mathrm{{{max}}}$) before failure; and midpoint strain ($\varepsilon _\mathrm{{{mid}}}$) or the strain at 50% of maximum stress. The diversity observed in the stress–strain curves corresponds to a high variability in the fitted parameters, detailed in Table S1 in SI. $E_1$ averaged 0.10 Mpa across the 24 human dermis skin samples, while $E_2$ averaged 20.59 Mpa. The $\sigma _\mathrm{{{max}}}$ gave an average value of 3.91 Mpa, and $\varepsilon _\mathrm{{{max}}}$ averaged around 38%. $\varepsilon _\mathrm{{{mid}}}$ exhibited a wide variability across the 24 human dermis samples, ranging from 0.18 to 0.46.

**Fig. 3:**
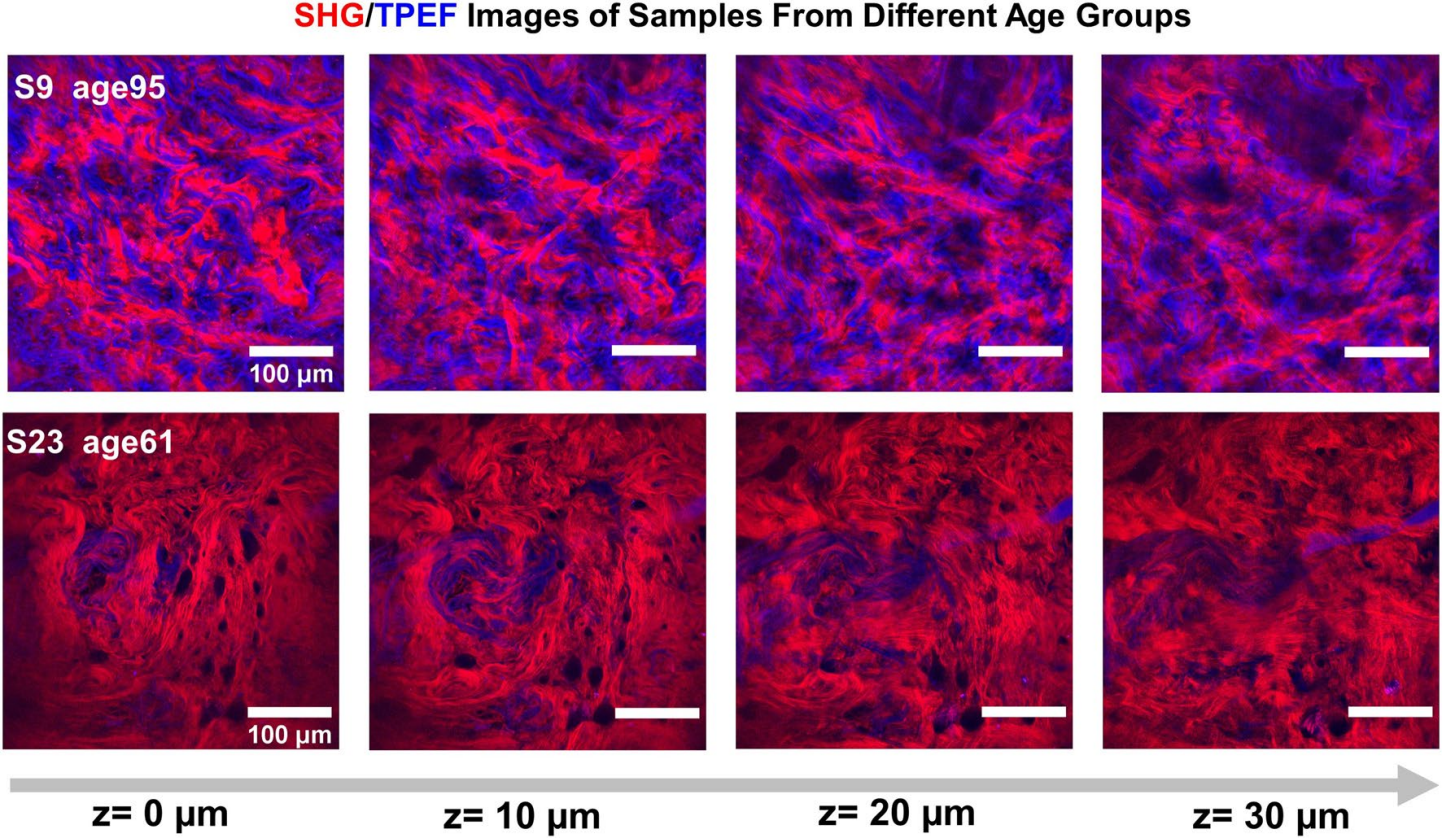
Pre-stretching images of collagen and elastin fibers of sample 9 and 23 at different depths. Collagen fibers in red color, elastin fibers in blue color. FOV: 400 $\times$ 400 $\upmu$m^2^. The presented images were processed with CLAHE. The intensity histograms of raw images and processed images of S9 and S23 at different imaging depths can be found in SI of Figure S2 and S3

### Structural analysis of collagen and elastin fibers

To investigate the structural factors underlying the stress–strain curves, we conducted an analysis of the 3D SHG/TPEF images of collagen and elastin fibers from each tissue sample before the stress–strain tests. Figure 3 shows the images of sample 9 and 23 from donors aged 95 and 61, respectively, with collagen fibers in red and elastin fibers in blue. We analyzed the orientation, density, and thickness of the collagen and elastin fibers in each 3D image stack of all samples. The measurement results show that the thickness of collagen fibers ranged from $16.7\pm 0.9$ to $111.8\pm 6.4\,\upmu$m, and that of elastin fibers ranged from $2.58\pm 0.06$ to $5.09\pm 0.24\,\upmu$m. The collagen densities of all samples ranged from 0.3 to 0.7, while the elastin densities of the samples ranged from 0.002 to 0.45. Further details of the measurement results of all samples can be found in Table S1, and the measurement illustration is shown in Figure S4.

**Fig. 4:**
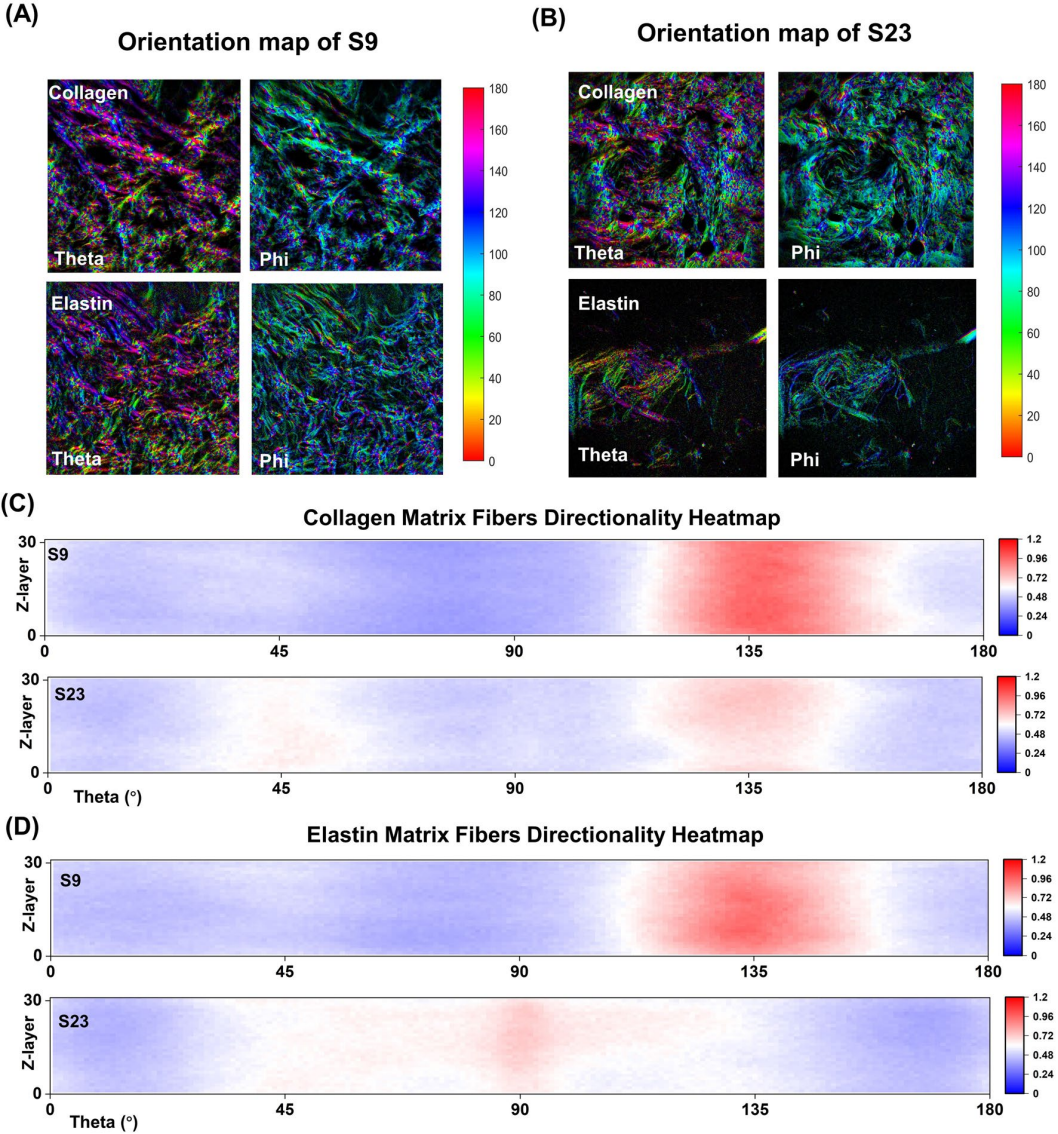
The orientation map of the collagen and elastin fibers of **A** sample 9, **B** sample 23 in the middle layer of the whole z-stack. Series of heatmaps represent the directional orientation of **C** collagen and **D** elastin fibers oriented between 1 and 180 degrees across the z-layer for 2 samples (S9, S23). The heatmap represents the orientation of collagen and elastin fiber as percentage of pixel count. As indicated by the scale bar on the right of each heatmap, the redder the color, the higher the counts. The z-layers are ranged from bottom to top in ascending order

Figure 4A and B shows the orientation map of the collagen and elastin fibers of S9 and S23. $\theta$ and $\varphi$ are the angles that define the fiber orientation in 3D space, with $\theta$ the angle in the *xy* imaging plane, and $\varphi$ the angle out of the plane (see Figure S5 A in SI, which presents a diagram of relevant angles, with the red line representing the main fiber orientation of the entire *z*-stack. $(\bar{C},\bar{S},\bar{Z})$ is the mass center, and the length of the red line is the value of 3DOI). An angle $\theta$ of $0^{\circ }$ and $180^{\circ }$ corresponds to the stretch direction, while a $\varphi$ angle of $90^{\circ }$ corresponds to in-plane orientation. To get insight into the fiber angle distribution across *z*-depth, we normalized the theta angle distribution for each layer and visualized it through a heatmap. The results reveal that there are two distinct families of fibers oriented at around $45^{\circ }$ and $135^{\circ }$ for S9 and S23 (Fig. 4C)). By summing and normalizing all layers within the *z*-stack, the representative collagen fiber distribution is obtained (See Figure S6 in SI for all angular distributions). Table 1 shows the variation of mean fiber angles and concentration parameters for 24 samples by modeling with a von Mises distribution. All samples show a strikingly similar $\theta$ distribution with two mean angles, peaking around $45^{\circ }$ and $135^{\circ }$, representing a consistent anisotropy of the samples in relationship to the stretch axis. To exclude experimental causes for this effect, i.e., resulting from the polarization state of the light, we first rotated the polarization state of the light from 0 to 60, 120 and 170 degrees and analyzed the orientation of the fiber bundles, see Figure S8 in SI. Clearly, changing the polarization angle leads to intensity differences, but the algorithm is capable of extracting a consistent set of fiber orientation parameters for each image (see Figure S9 for the fiber angular distribution for various polarization states of light). We next compared the orientation distribution of a sample that was placed in the stretcher in the usual way and after a $90^{\circ }$ rotation of the images (See Figure S10). Rotating the image correspondingly alters the peak of the theta distribution into a distribution complementary to the original. Therefore, we conclude that the specific distribution of theta angles for our tissue samples arises from the consistently used cutting procedure of the tissue from the thigh. We hypothesize that the orientation of the collagen fibers with respect to the cutting direction and the stretch axis, is due to the body’s tension lines, also called Langer’s lines. In order to emphasize the angle between the stretch axis and the main orientation of the fibers, we transformed all main angles $\theta$ within the ($0^{\circ }\,180^{\circ }$) range into the ($0^{\circ }\,90^{\circ }$) range, with angles greater than $90^{\circ }$ adjusted to ($180^{\circ }$ - $\theta$). The main fiber angle of all samples falls in the range $(27^{\circ } \sim 49.5^{\circ })$ (See Table S1 in SI). Figure S5B shows four examples of main $\theta$ angle of collagen fibers.

**Table 1:** Variation of main fiber angles ($\alpha _1$,$\alpha _1$) and concentration parameters ($a_1$, $a_2$) across 24 samples by modeling with von Mises distribution

Sample: human thigh dermis skin; Thickness: 0.8 mm												
Sample Information			Collagen fiber					Elastin fiber				
No	Gender	Age	*w*	$\alpha _1 (^{\circ })$	$a_1$	$\alpha _2 (^{\circ })$	$a_2$	*w*	$\alpha _1 (^{\circ })$	$a_1$	$\alpha _2 (^{\circ })$	$a_2$
S1	Male	77	0.57	31.83	0.51	135.35	1.94	0.58	40.96	0.71	134.71	1.05
S2	Male	84	0.76	0	0.34	138.45	3.38	0.86	1.47	0.35	137.44	4.06
S3	Female	87	0.47	42.55	2.39	138.87	0.63	0.21	43.10	3.26	179.99	0.27
S4	Male	79	0.53	46.77	0.56	134.29	2.10	–	–	–	–	–
S5	Female	88	0.61	0	0.37	138.77	2.84	0.76	10.07	0.22	135.51	2.50
S6	Male	82	0.52	51.42	1.07	129.71	1.66	0.86	60.07	0.25	132.05	4.22
S7	Female	90	0.47	39.20	2.07	150.85	0.76	0.96	34.12	0.26	136.47	7.47
S8	Female	87	0.53	50.93	0.60	132.37	2.11	0.91	110.08	0.16	134.27	6.90
S9	Male	95	0.32	37.24	1.20	137.51	2.00	0.49	34.23	1.41	152.05	0.79
S10	Male	83	0.57	0	0.17	134.58	3.67	0.77	0	0.27	135.65	3.35
S11	Male	66	0.48	43.87	1.32	141.04	1.04	0.94	83.28	0.14	135.53	4.98
S12	Female	88	0.64	15.42	1.37	150.57	2.68	0.73	16.37	1.67	149.96	2.36
S13	Female	94	0.48	49.94	1.04	128.04	1.66	0.34	47.94	1.46	126.41	0.62
S14	Female	75	0.51	43.32	0.69	141.20	1.41	0.89	18.01	0.38	138.89	3.84
S15	Male	82	0.51	12.80	1.02	145.40	2.34	0.67	0	0.99	145.82	1.77
S16	Male	75	0.62	23.54	0.64	137.05	1.98	0.32	41.33	1.31	146.98	0.51
S17	Female	88	0.54	22.98	1.36	149.17	1.97	0.39	26.14	1.36	157.74	1.17
S18	Female	90	0.38	36.48	2.24	150.42	1.05	0.01	0.61	63.34	95.24	0.32
S19	Male	79	0.47	43.65	3.64	180	0.52	0.39	41.87	1.26	149.95	0.34
S20	–	84	0.61	41.52	0.85	135.59	1.67	–	–	–	–	–
S21	Female	85	0.57	11.65	0.40	139.67	2.23	0.76	0	0.52	145.97	1.20
S22	Female	88	0.48	21.95	1.71	154.23	1.42	0.64	36.02	0.45	136.10	1.84
S23	–	61	0.51	47.16	0.93	134.48	1.32	0.07	42.64	4.24	95.54	0.38
S24	Female	85	0.51	41.94	0.79	139.78	1.77	0.63	34.07	0.76	146.31	1.46

– indicates the information was lost or the parameter was hard to determine

The $\theta$ distribution of elastin fibers over the *z*-direction shows a similar distribution to that of collagen fibers, although the primary angles are not consistent at around $45^{\circ }$ and $135^{\circ }$ (Fig. 4D). See Figure S7 for all representative theta angular distribution of elastin fibers, and detailed von Mises distribution modeling parameters are available in Table 1.

To quantify the orientation in each tissue sample prior to stretching, we calculated the orientation index, which represents the overall orientation of fibers in a *z*-stack and ranges from 0 for strongly disordered to 1 for highly aligned fibers. In our images, the 3DOI of the collagen ranges from 0.06 to 0.25, while for the elastin ranges from 0.01 to 0.24, as shown in Table S1.

**Fig. 5:**
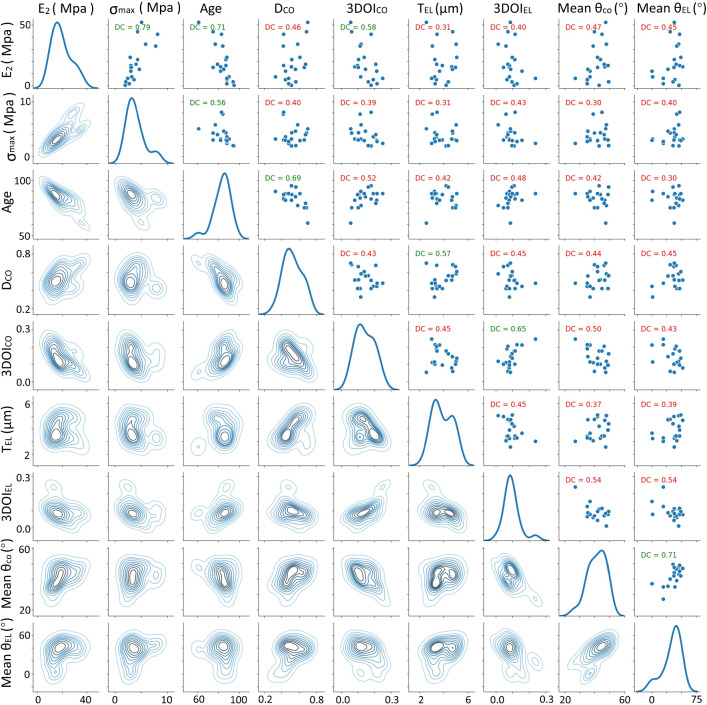
The upper half of the diagram shows the scatter plots between the various variables and the distance correlation in green ($p < 0.05$) and red ($p > 0.05$). On the diagonal the kernel density estimates of the single variable distributions are shown. The lower half of the diagram shows the isolines of the kernel density estimates of the various bivariate distributions. The distributions and correlations between all variables can be found in SI (Figure S3).$E_2$: Elastic Young’s modulus, $\sigma _\mathrm{{{max}}}$ maximum stress, age of the donor; $D_{CO}$, density of collagen fibers; $3DOI_{CO}$, collagen 3D orientation index; $T_{EL}$, elastin fiber thickness; $3DOI_{EL}$, elastin 3D orientation index; main $\theta _{CO}$, main $\theta$ angle of collagen fibers, main $\theta _{EL}$, and main $\theta$ angle of elastin fibers

**Fig. 6:**
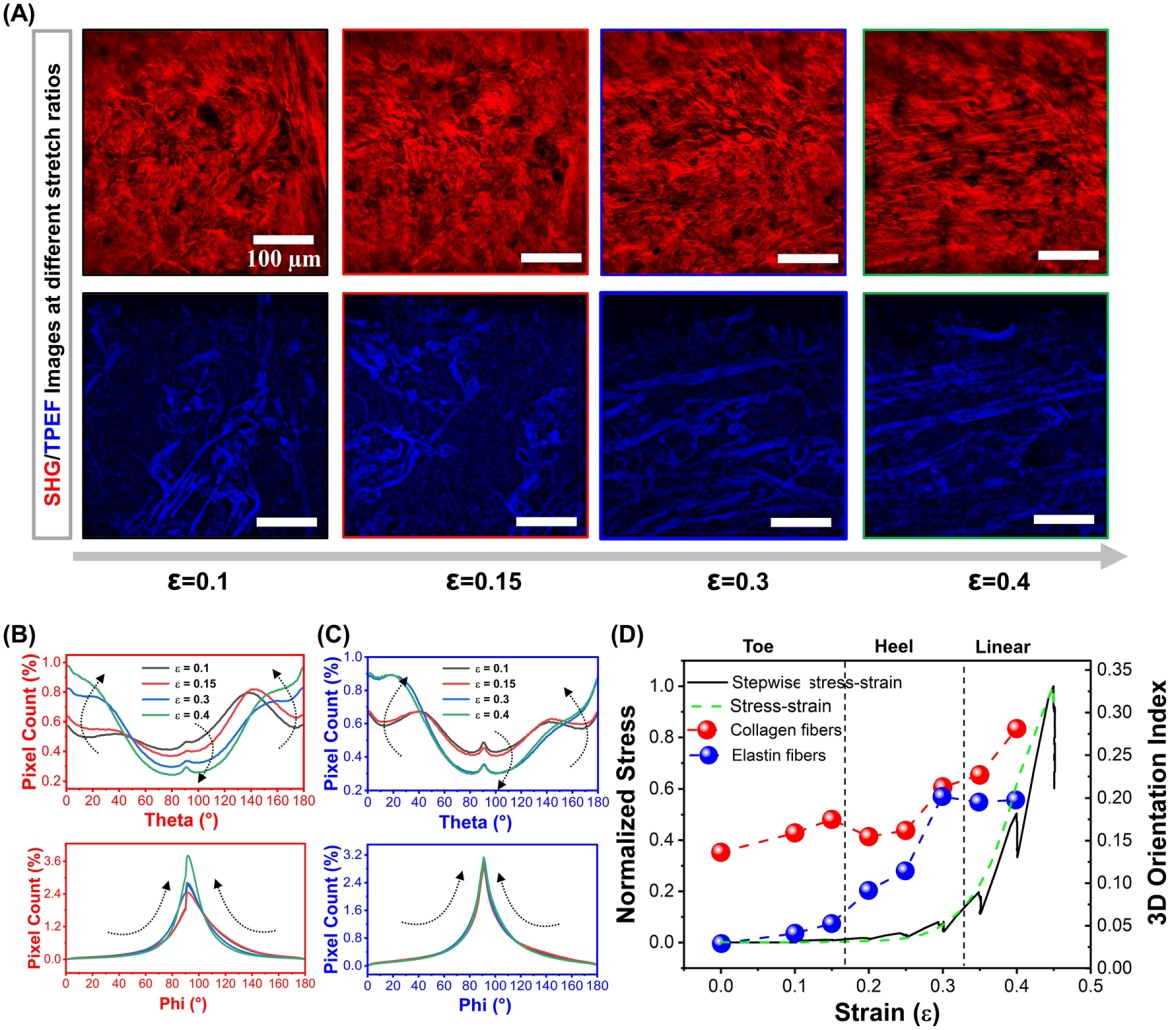
Evolution of collagen and elastin fiber orientation of a human thigh dermis skin sample during stretching (sample information: female, age 88, S22). **A** Representative 2D images of collagen fibers (first row) and elastin fibers (second row) at different strain, which are extracted from corresponding 3D image stacks. FOV: $400\times 400\,\upmu$m^2^ (clip off dark invalid areas around the edges from a $500\times 500\,\upmu$m^2^ raw data). 3D image stacks at different strain can be seen in the SI (visualization 1, 2, 3 and 4 for $\varepsilon$ = 0.1, 0.15, 0.3 and 0.4, respectively), and a time-lapse video monitoring the changes of the fibers during stretching can be seen in visualization 5. $\theta$ and $\varphi$ angular distribution of **B** collagen and **C** elastin fibers across the *z*-stack at different strain. **D** The 3DOI of collagen and elastic fibers with increasing strain, and the corresponding stress–strain curves, with solid black line indicating the stress–strain curve with stops for imaging, and the dashed green line for the stress–strain curve measured prior to imaging at a continuous stretch speed of 0.5 mm/s. The dashed black lines indicate the toe, heel, and linear regions (Fratzl et al. 1998; Gutsmann et al. 2004)

### Microstructural factors and mechanical properties correlations

To investigate which microstructural factors most underlie the mechanical properties of the skin tissue, we conducted a comprehensive analysis of the correlation between five key characteristics of mechanical properties ($E_1$, $E_2$, $\sigma _\mathrm{{{max}}}$, $\varepsilon _\mathrm{{{max}}}$, $\varepsilon _\mathrm{{{mid}}}$) and pre-stretching microstructural properties: density (D), fiber thickness (T), 3DOI, and the main $\theta$ angle for both collagen (subscript CO) and elastin (subscript EL). The age of the donors was also included in this analysis. Pearson’s R coefficient only captures linear dependencies between variables. To also capture (possible) nonlinear dependencies between the five characteristics and the microstructural properties, we calculated the so-called distance correlation (DC) between the various variables (Székely et al. 2007; Székely and Rizzo 2014). The intuition between the distance correlation is the following: if for two random vectors *X* and *Y* the matrix of pairwise distances between observations from *X* and the analogous distance matrix for observations from *Y* co-vary together, we say that *X* and *Y* have a large distance correlation. If they do not, they have a small distance correlation. The distance correlation ranges between 0 and 1. To calculate the significance of the dependency, we used bootstrapping (re-sampling the data 2000 times) to calculate the p-value. Both distance correlation and significance were calculated with the Python package dcor (https://github.com/vnmabus/dcor).

This gives us a dataset of 24 samples each having 13 features (data columns). Of the 24 samples measured, some samples contained missing data. Three samples had missing $E_1$ values, and two samples had missing elastin properties (D, T, 3DOI, and main $\theta$ angle). It can be good practice to impute missing data in a column with, e.g., the mean of a specific data column. However, in our case due to the small sample size we decided to drop the data rows with missing data. The data set now contains 19 samples.

**Fig. 7:**
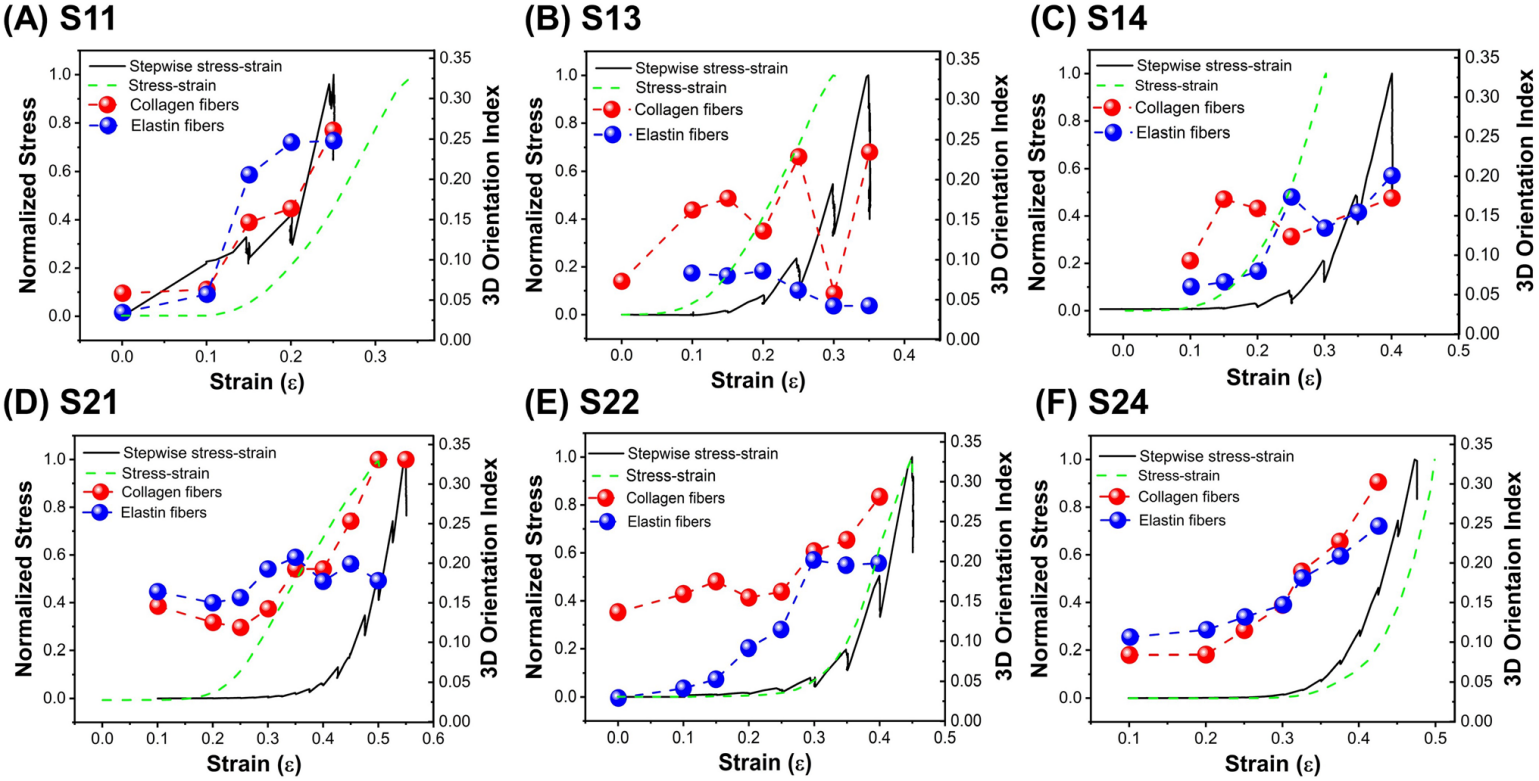
3DOI of collagen and elastin fibers with dynamic stress–strain curves. First row is low-strain midpoint group, **A** S11, **B** S13, **C** S14. Second row is high-strain midpoint group **D** S21, **E** S22, **F** S24

**Fig. 8:**
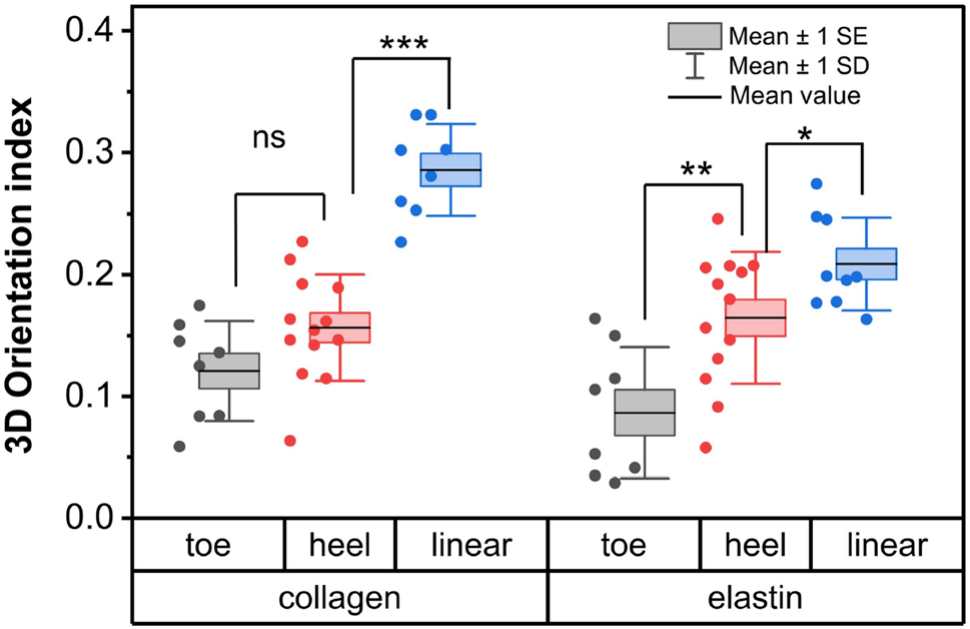
The box plot of mean value of 3DOI at different stress regions of collagen and elastin fibers. * stands for $p<0.05$, ** for $p< 0.01$, *** for $p< 0.001$ and ns for no significant difference

The distributions and correlations between all variables can be found in SI (Figure S11), and a selection of the results is displayed in Fig. 5. The upper half of the diagram shows the scatter plots between the various variables. A cut-off value of $p=0.05$ was used to indicate whether the dependency was statistically significant. Green denotes significant results ($p<0.05$), red indicates that the dependencies were found to be not significant ($p > 0.05$). The diagonal shows the various univariate distributions, smoothed out by kernel density estimation. The lower half of the diagram shows the isolines of the kernel density estimations of the various bivariate distributions.

Age was found to correlate well with both $E_2$ and $\sigma _\mathrm{{{max}}}$ with distance correlation $DC = 0.71$ and $DC = 0.56$ respectively. From the scatter plots we can see that both characteristics decrease with increasing age. Age also showed a negative dependency on the collagen density $D_{CO}$ ($DC = 0.69$). The overall collagen orientation index $3DOI_{CO}$ increases with increasing age ($DC=0.52$). $E_2$ showed a negative correlation with the collagen orientation index $3DOI_{CO}$ ($DC = 0.58$). The pairs $3DOI_{CO}$ and $3DOI_{EL}$ as well as main $\theta _{CO}$ and main $\theta _{EL}$ show a strong positive dependency (DC = 0.65 and 0.71, respectively) suggesting that both fiber structures co-align to a high degree. The density of collagen fibers $D_{CO}$ shows a positive correlation (DC = 0.57) with $T_{EL}$, the elastin thickness, suggesting that tissue with high collagen density is associated with thicker elastin fibers. Elastin density, collagen thickness, and $E_1$ showed no significant correlations ($DC < 0.5$) with any of the other variables.

### Dynamic imaging during stretching

To visualize the real-time changes of the microstructures during stress, we conducted a stepwise stretching of the sample at a 0.05 strain. After each step, we shortly paused the motors for SHG/TPEF imaging. We present the results of sample 22 in Fig. 6, and the 3D image stacks at different strains can be found in SI (visualizations 1-4), while a time-lapse video is available in visualization 5. The angular distributions of the collagen and elastin fibers at different strains were analyzed for the full *z*-stack, and the $\theta$ and $\varphi$ angular distributions of sample 22 are shown in Fig. 6B and C, respectively. Figure 6D shows the 3DOI of collagen and elastin fibers at the different strains plotted on the corresponding stress–strain curves. Note that during the imaging time, some relaxation occurred, as indicated by the drops in the stress–strain curve which could affect the mechanical response. The images are therefore representative for a certain region in the stress strain curves. In the toe region, from $\varepsilon$ = 0.1 to 0.15, already some alignment is observed, but the fibers are still quite randomly oriented. The distribution of the $\theta$ and $\varphi$ angles increases slightly toward $0^{\circ }$ and $180^{\circ }$, and $90^{\circ }$, respectively, with the 3DOI increasing from 0.18 to 0.19 (Fig. 6B, D). The dominant $\varphi$ angle is $90^{\circ }$, corresponding to an in-plane orientation, which may be due to the limited reference in the z-direction. In the heel region ($\varepsilon$ = 0.3), the collagen fibers begin to align with the stretch direction, and the 3DOI increases to 0.22. The elastin fibers are aligned at this strain already. In the linear region at $\varepsilon$ = 0.4, the images show that the collagen fibers have aligned, appear to be closer together and bundled. The 3DOI of the collagen fibers increases from 0.18 to 0.24 in the heel region, and to 0.3 in the linear region (Fig. 6A, D). The elastin fibers appear to align at lower stress and are fully aligned at a level of 0.23 in the heel region.

We were able to observe the same ROI for the entire stress–strain measurement 6 times. Consistent with the variation observed in the stress-strain curves in Fig. 2, the behavior of the collagen and elastin orientation index as a function of strain varied for each measurement. We grouped the data into two categories based on the midpoint strain, with $\varepsilon _\mathrm{{{mid}}} >0.27$ (low-strain midpoint group, first row, S11, S13, S14) and $\varepsilon _\mathrm{{{mid}}}>0.27$ (high-strain midpoint group, second row, S21, S22, S24), and plotted the stress–strain curves and orientation indices for all 6 experiments in Fig. 7. The data of S13 and S14 are problematic as almost no alignment is observed. However, from the other curves it appears that all start out with a lower orientation index for both collagen and elastin and that the 3DOI of collagen fibers starts to increase in the heel region, followed by a further increase in the linear region. The drops in collagen 3DOI within the heel region for S21 and S22 suggest an influence from the samples’ anisotropic properties. When collagen fibers within an anisotropic sample undergo reorientation in the stretch direction, it initially results in a decreased orientation index, followed by eventual alignment in the stretch direction. A similar trend was observed in a study by Witte et al. (2021), comparing stretching behavior in isotropic and anisotropic samples. They found that collagen fibers within anisotropic samples exhibited an initial tendency to align themselves before predominantly orienting on the stretching plane, similar to the current observation for the two samples with relatively high initial 3DOI. In 3 out of 4 cases elastin orientation occurred before collagen orientation. To provide overarching conclusions regarding the structural changes underlying the stress–strain curve, we performed a statistical analysis of the mean value of the 3DOI in the three different stress regions (toe, heel, and linear) for four experiments (S11, S21, S22, and S24) in Fig. 8. We found that the 3DOI of collagen fibers significantly increased only in the linear region, while the 3DOI of elastin fibers significantly increased in both the heel and linear regions.

## Discussion

In this study, we utilized a custom-built skin stretch device equipped with label free SHG and TPEF imaging, to record microstructural dynamic images in human dermis skin samples from the thigh under uniaxial stretching. To better direct the design of biomimetic materials and induce skin regeneration in wounds with more optimal outcome and insight into healthy skin maintenance, ideally one would like to be able to model skin mechanical properties from its structural components. As a step toward this goal, here, we collected a comprehensive dataset of 24 subjects ranging in age, though skewed toward the elderly range, displaying a wide variation in stress–strain curves. To investigate which particular microstructural features correlate with the main mechanical properties, we performed an analysis of the correlation among five key mechanical properties, pre-stretching microstructural properties and age, by calculating the distance correlation (DC) between the various variables.

We find that age correlates negatively with Young’s modulus ($E_2$), maximum stress ($\sigma _\mathrm{{{max}}}$) and collagen density, and positively with collagen orientation index. In other words, the Young’s modulus shows a trend to decrease with age, from 36 MPa at age 61, to 10 MPa at age 95, illustrating that the ability of skin to resist elastic elongation is lost with age (from 60 to 100 years old). This result is in line with in vivo research (Pailler-Mattei et al. 2013; Komatsu et al. 2004), though some studies have reported the opposite conclusion (Diridollou et al. 2001; Escoffier et al. 1989). The reported values for the Young’s modulus of skin depend on the model used and the stress applied and range from 0.02 MPa to 57 MPa (Pailler-Mattei et al. 2013), illustrating the difficulty of extracting this parameter from in vivo measurements. Most likely, the studies reporting a low Young's modulus have been limited to what we report here as the toe region, as the low range reported values agree with the $E_1$ value of 0.1MPa (Table S1 in SI).

The negative correlation of collagen density with age may be due to remodeling-type processes becoming dominant, as collagen synthesis decreases, eventually resulting in the loss of density of collagen bundles (Marcos-Garcés et al. 2014). The increase of collagen 3DOI with age suggests a structural change in the collagen matrix with aging that goes beyond loss of fibers. Because of the correlation with age, we can also state that collagen density correlates with Young’s modulus, even if the direct DC between $E_2$ and collagen density is only 0.46. This may be expected, as it means that more collagen leads to a stiffer material with higher $E_2$. $E_2$ showed a negative correlation (DC 0.58) with the orientation index of collagen fibers and has a low, not significant, correlation (DC 0.47) with the main $\theta _{CO}$.

The collagen and elastin orientation indices $3DOI_{CO}$ and $3DOI_{EL}$ as well as the main $\theta _{CO}$ and main $\theta _{EL}$ each show a strong positive dependency (DC = 0.65 and 0.71, respectively) suggesting that both fiber structures co-align to a high degree. Also, the density of collagen fibers $D_{CO}$ shows a positive correlation (DC = 0.57) with $T_{EL}$, the elastin thickness, suggesting that tissue with high collagen density is associated with thicker elastin fibers.

The dynamic stress–strain curves were affected by relaxation phenomena during the imaging time (Pukaluk et al. 2023). Nevertheless, the dynamic imaging under certain regions of strain experiments show that the 3D orientation index of collagen fibers increased continuously with increasing strain, and that the maximal 3DOI of the elastin fibers was reached before that of the collagen fibers. The orientation index of collagen fibers increased significantly only in the linear region, while the value of elastin fibers increased significantly in both the heel and linear regions. This shows that at lower strain (toe and heel region), the force is strong enough to engage and orient the elastin fibers, but not strong enough to engage the collagen fibers. Therefore, the elastin fibers play a main role in withstanding the deformation of skin at lower strain. When the strain increases to a certain value (linear region), the collagen fibers begin to take on the main force, and correspondingly, the same strain increment in this region requires a larger stress than before. In other words, collagen and elastin networks can deform independently with a certain degree of freedom. This discovery aligns with findings presented in Holzapfel et al. (2015), but break the common assumption of affine deformation. Affine deformation simplifies fiber-based mechanical modeling and might not hold true in complex biological tissues or materials where nonlinear behaviors, fiber interactions, or variations in local deformations exist. The von Mises modeling with the angle distribution of collagen and elastin fibers in our work will be helpful for incorporating more complex behavior to build an advanced model of soft tissues.

There are several limitations to this study that warrant attention in subsequent research:

- Due to the limited number of the samples and the skewed age group with subjects of 60 to 91 years old examined in this study, some correlation between microstructurals and the mechanical parameters may not have arisen. However, the difficulty in obtaining and testing samples from younger donors limits further examination. Ideally, a follow-up study should involve a broader age group and a larger cohort, so that the step can be made to modeling and predicting the mechanical properties from the microstructural features. Furthermore, a follow-up study should include tissue cut with different orientation from the donors thigh, to generate a wider fiber main angle distribution and thereby investigate the correlation between fiber orientation with mechanical parameters better.
- We utilized split-thickness skin in this study, ensuring uniform thickness across the sample, thus enabling homogeneous in-plane tension and allowing examination at a specific depth of the skin. However, our measurements were limited to a depth of 30 $\upmu$m. Subsequent studies should expand the depth of measurement and consider correlating analysis across various depths.
- While we modeled the fiber angle distribution using the von Mises distribution, our study did not encompass tissue modeling for estimating mechanical parameters. Consequently, a subsequent study should address this concern.
- We focused on characterizing the uni-directional mechanical properties of human thigh skin. The next step could involve conducting bi-directional experiments to delve deeper into exploring the skin’s anisotropic properties.

## Conclusion

Overall, this study provides valuable insights into the mechanical properties of human dermal skin and highlights the importance of the collagen and elastin fiber density and alignment for the skin elastic properties. The acquisition of a comprehensive dataset and the modeling of fiber angle distribution using the von Mises distribution present valuable parameters for tissue modeling. Moreover, a comprehensive analysis of the correlation between the mechanical properties and micro structural properties are achieved. This study further established that the collagen and elastin content changes with age and how this affects the skin mechanical properties. A comprehensive understanding of these complex interconnections among skin structure, mechanical behavior, and aging holds profound implications for skin health, disease, material modeling, as well as clinical and biomechanical applications.

### Supplementary Information

Below is the link to the electronic supplementary material.

Supplementary file 1 (pdf 20690 KB)

Supplementary file 2 (avi 1686 KB)

Supplementary file 3 (avi 1779 KB)

Supplementary file 4 (avi 1714 KB)

Supplementary file 5 (avi 1151 KB)

Supplementary file 6 (avi 1747 KB)
