# Supplementary material for: Uniaxial mechanical stretch properties correlated with three-dimensional microstructure of human dermal skin
Source: Biomech Model Mechanobiol. 2024 Feb 7;23(3):911–25. doi: 10.1007/s10237-023-01813-3 (PMC11101527; doi:10.1007/s10237-023-01813-3)
Supplement: Supplementary file 1 — (pdf 20690 KB) [file 10237_2023_1813_MOESM1_ESM.pdf]

Supplementary Information for "Uniaxial mechanical stretch properties correlated with three-dimensional microstructure of human dermal skin"

Mengyao Zhou[1*†], Patrick José González[1†], Ludo Van Haasterecht[1,2,3], Alperen Soylu[1], Maria Mihailovski[1], Paul Van Zuijlen[2,3,4,5], Marie Louise Groot[1]

[1*]Faculty of Science, Department of Physics, Laserlab, Vrije Universiteit Amsterdam, De Boelelaan 1105, 1081HV, Amsterdam, The Netherlands.

[2]Burn Center and Department of Plastic, Reconstructive and Hand Surgery, Red Cross Hospital, Mozartstraat 201, 1962 AB, Beverwijk, The Netherlands.

[3]Department of Plastic, Reconstructive and Hand Surgery , Amsterdam University Medical Center (UMC), Location Vrije Universiteit Amsterdam, De Boelelaan 1117, 1081 HV, Amsterdam, The Netherlands.

[4]Pediatric Surgical Centre, Emma Children's Hospital, Amsterdam University Medical Center (UMC), Location University of Amsterdam, Meibergdreef 9, Amsterdam, The Netherlands.

[5]Amsterdam Movement Sciences (AMS) Institute, Amsterdam University Medical Center (UMC), Location Vrije Universiteit Amsterdam, Meibergdreef 9, Amsterdam, The Netherlands.

*Corresponding author(s). E-mail(s): m.zhou@vu.nl;
†These authors contributed equally to this work.

Supplementary table

Table S1 Summary of the measurement results of 24 samples

Sample: human thigh dermis skin; Type: Ex-vivo uniaxial stretch; Thickness: 0.8 mm

Sample Information			Mechanical measurements					Collagen fibers				Elastin fibers			
**No.**	**Gender**	**Age**	$E_1(Mpa)$	$E_2(Mpa)$	$\sigma_{max}(Mpa)$	ε_{max}	ε_{mid}	**D**	**T (μm) (±SE)**	**3D OI**	**Main θ (0° 90°)**	**D**	**T (μm) (±SE)**	**3D OI**	**Main θ (0° 90°)**
S1	Male	77	0.50	27.14	5.84	0.30	0.18	0.58	51.4±2.7	0.12	48°	0.02	5.04±0.19	0.01	45°
S2	Male	84	0.13	26.35	7.76	0.42	0.27	0.42	45.4±4.1	0.11	39°	0.09	3.06±0.14	0.06	42°
S3	Female	87	0.12	20.84	4.32	0.34	0.23	0.52	53.2±3.7	0.10	44°	0.03	3.27±0.14	0.11	38°
S4	Male	79	0.17	17.13	3.22	0.30	0.19	0.59	35.8±4.0	0.10	45°	–	–	–	–
S5	Female	88	0.20	12.50	3.58	0.45	0.26	0.44	48.1±2.2	0.20	35°	0.12	3.41±0.22	0.07	39°
S6	Male	82	0.07	17.88	3.18	0.31	0.21	0.51	34.7±3.5	0.08	47°	0.04	4.67±0.27	0.08	52°
S7	Female	90	0.03	13.70	3.18	0.38	0.24	0.33	42.9±3.3	0.12	37°	0.002	3.25±0.15	0.08	1°
S8	Female	87	0.19	18.31	3.01	0.33	0.26	0.42	111.8±6.4	0.10	49°	0.03	3.90±0.17	0.08	47°
S9	Male	95	0.06	10.25	1.91	0.30	0.18	0.51	35.8±2.6	0.18	43°	0.45	4.20±0.08	0.11	46°
S10	Male	83	0.09	31.07	8.09	0.42	0.27	0.42	52.9±2.5	0.18	45°	0.06	3.45±0.19	0.12	47°
S11	Male	66	–	30.11	5.52	0.35	0.26	0.48	19.7±0.6	0.08	35.7°	0.09	3.59±0.10	0.04	43.5°
S12	Female	88	0.04	13.03	2.39	0.34	0.22	0.48	32.5±2.1	0.25	27°	0.15	3.01±0.16	0.24	19°
S13	Female	94	0.08	11.60	2.01	0.30	0.22	0.56	68.3±4.2	0.16	49.5°	0.22	4.39±0.09	0.10	35.5°
S14	Female	75	0.07	32.20	4.31	0.30	0.25	0.68	58.8±3.0	0.06	40°	0.19	4.74±0.18	0.07	31°
S15	Male	82	0.06	16.06	2.98	0.33	0.23	0.44	38.5±3.2	0.21	37°	0.07	3.67±0.15	0.16	0°
S16	Male	75	0.08	17.54	3.05	0.34	0.25	0.66	44.0±1.4	0.10	42°	0.05	4.98±0.15	0.04	37°
S17	Female	88	0.10	10.91	2.72	0.44	0.31	0.48	43.1±2.0	0.22	35°	0.09	3.30±0.17	0.13	19°
S18	Female	90	–	13.14	3.39	0.43	0.31	0.52	16.7±0.9	0.13	42.7°	0.04	4.44±0.16	0.07	90°
S19	Male	79	0.08	18.90	3.98	0.39	0.29	0.68	32.0±2.1	0.21	46.5°	0.02	3.43±0.18	0.07	41°
S20	–	84	0.05	16.24	2.77	0.39	0.30	0.56	45.2±2.2	0.07	45°	–	–	–	–
S21	Female	85	0.02	16.81	4.65	0.50	0.35	0.56	45.1±1.4	0.15	35.4°	0.24	4.73±0.11	0.09	27°
S22	Female	88	0.001	21.66	2.90	0.45	0.39	0.61	47.3±1.7	0.14	42°	0.23	5.09±0.24	0.10	49°
S23	–	61	0.01	35.99	5.08	0.50	0.45	0.70	49.4±1.9	0.06	47.5°	0.05	2.58±0.06	0.09	38°
S24	Female	85	–	44.80	4.02	0.50	0.46	0.63	42.8±2.2	0.08	42°	0.22	4.61±0.25	0.09	32°

Note: – indicate the information was lost or the parameter was hard to determine.
SE (Standard Error)
D: density
T: thickness

Supplementary Figures

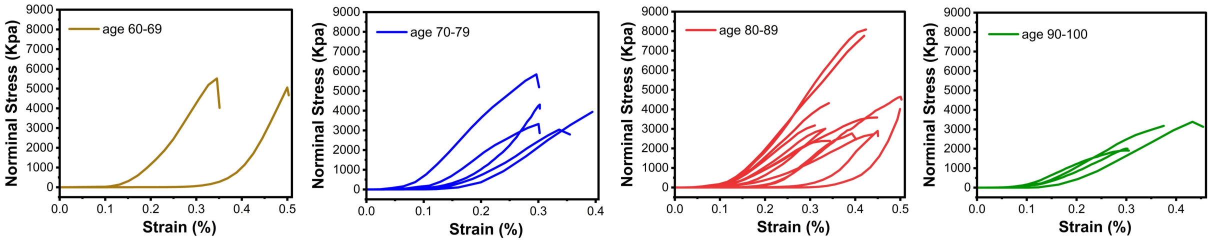

Fig. S1 Stress-strain curves of 24 samples with different color indicate different age group.

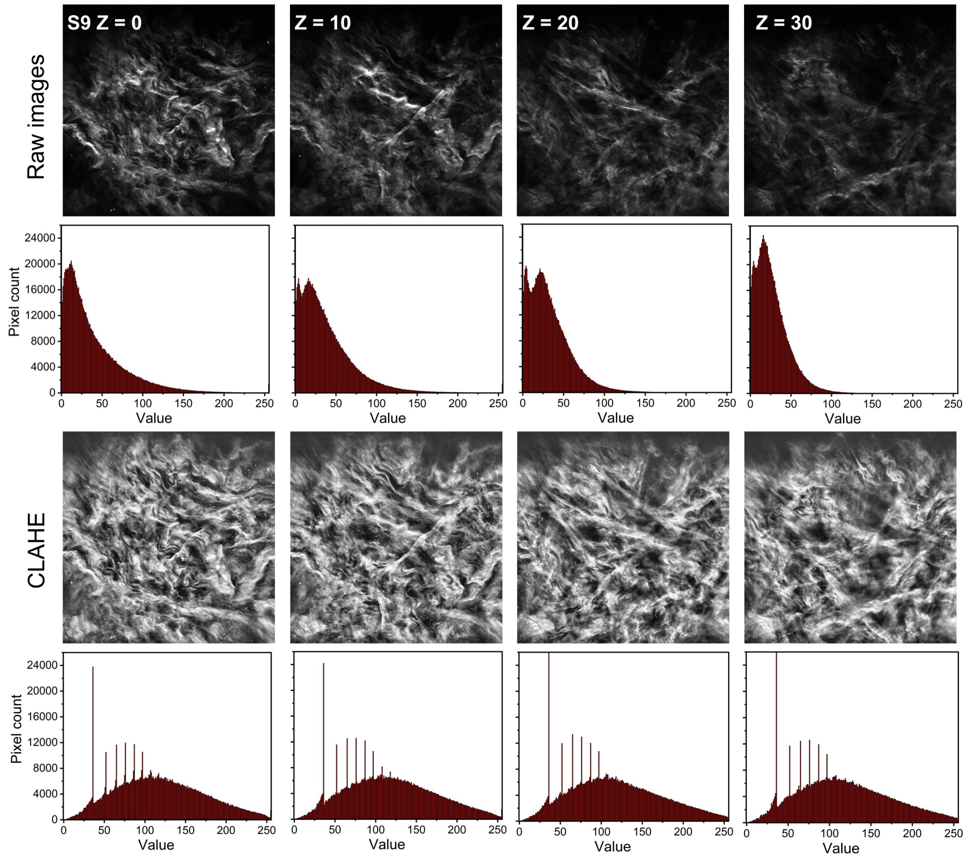

Fig. S2 The intensity histograms of raw images and processed images with CLAHE (enhanced using contrast limited adaptive histogram equalization) of s9 at defferent imaging depth. FOV: 500×500 μm^2.

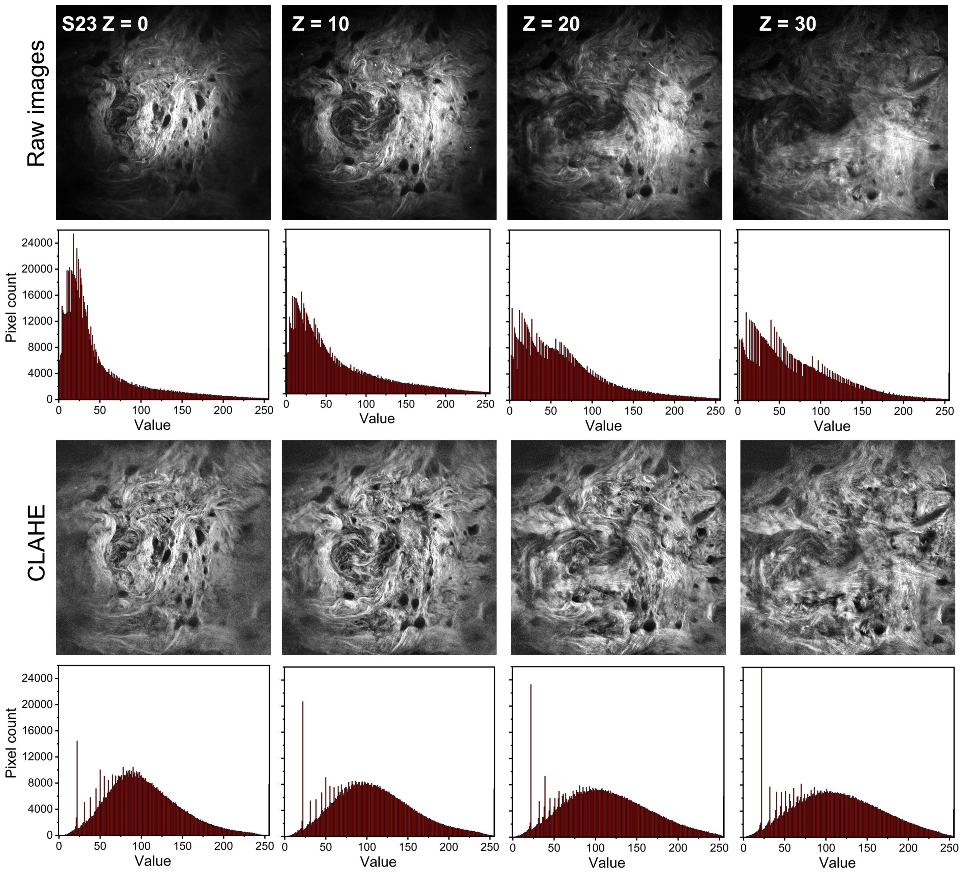

Fig. S3 The intensity histograms of raw images and processed images with CLAHE (enhanced using contrast limited adaptive histogram equalization) of s23 at defferent imaging depth. FOV: 500 × 500 μm^2.

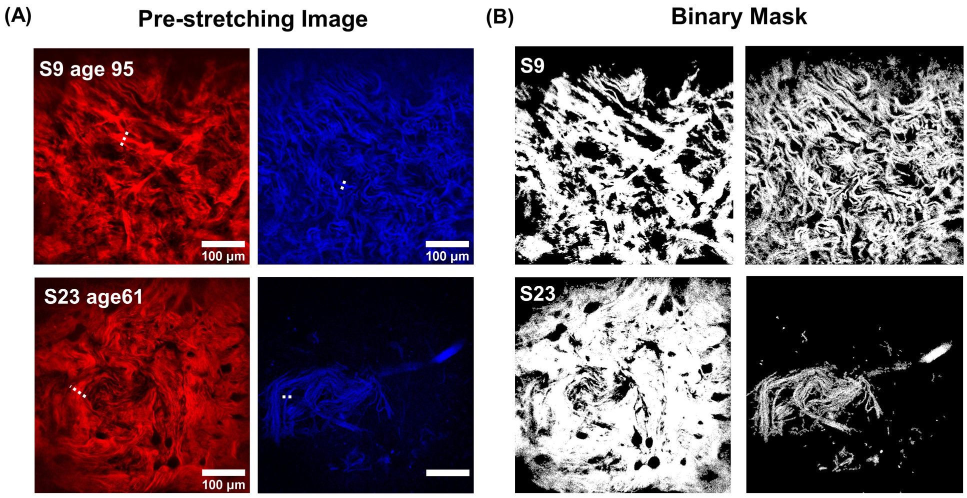

Fig. S4 (A) Pre-stretching images of collagen and elastin fibers of sample 9 and 23, with white dashed lines indicating an example of the measured fiber thickness. Collagen fibers in red color and elastin fibers in blue color. (B) Binary masks of collagen and elastin fibers for those two samples. FOV: 400 × 400 μm^2. The presented images are the middle layer images of the whole z stack.

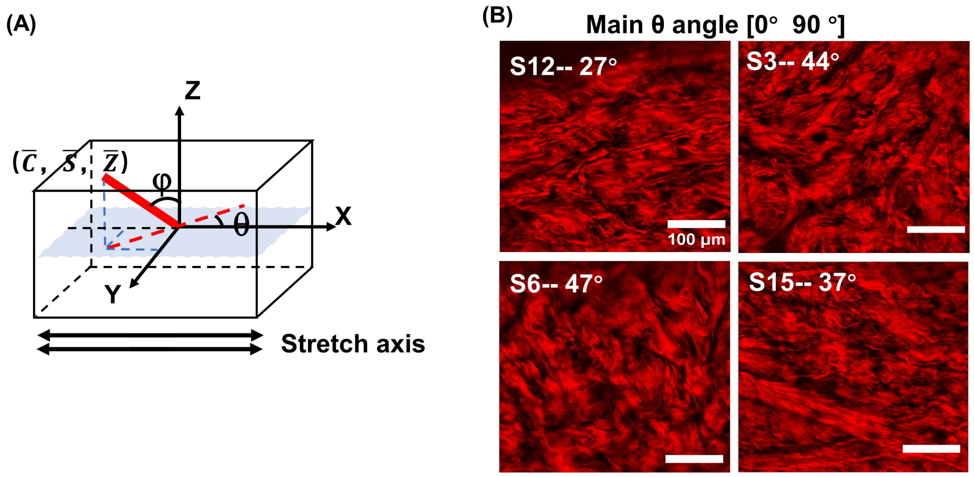

Fig. S5 (A) Angle diagram. Red line represents the main of fiber orientation of the entire z-stack. $(\bar{C}, \bar{S}, \bar{Z})$ is the mass center. The length of this red line represents the overall orientation of fibers in the z-stack. (B) The main θ angle of four samples. FOV: 400 × 400 μm^2

.

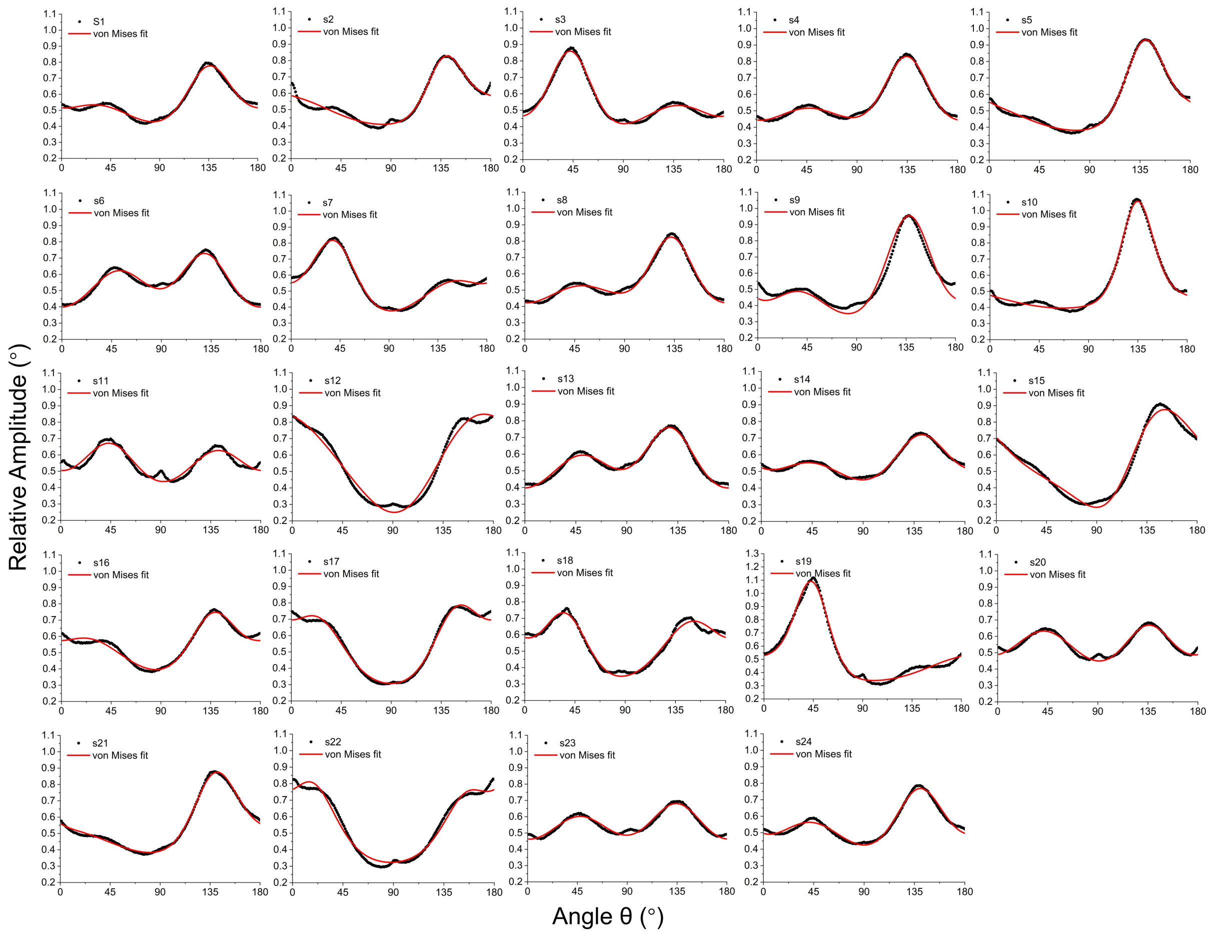

Fig. S6 Representative collagen fiber distributions of 24 samples fitted with von-Mises distribution.

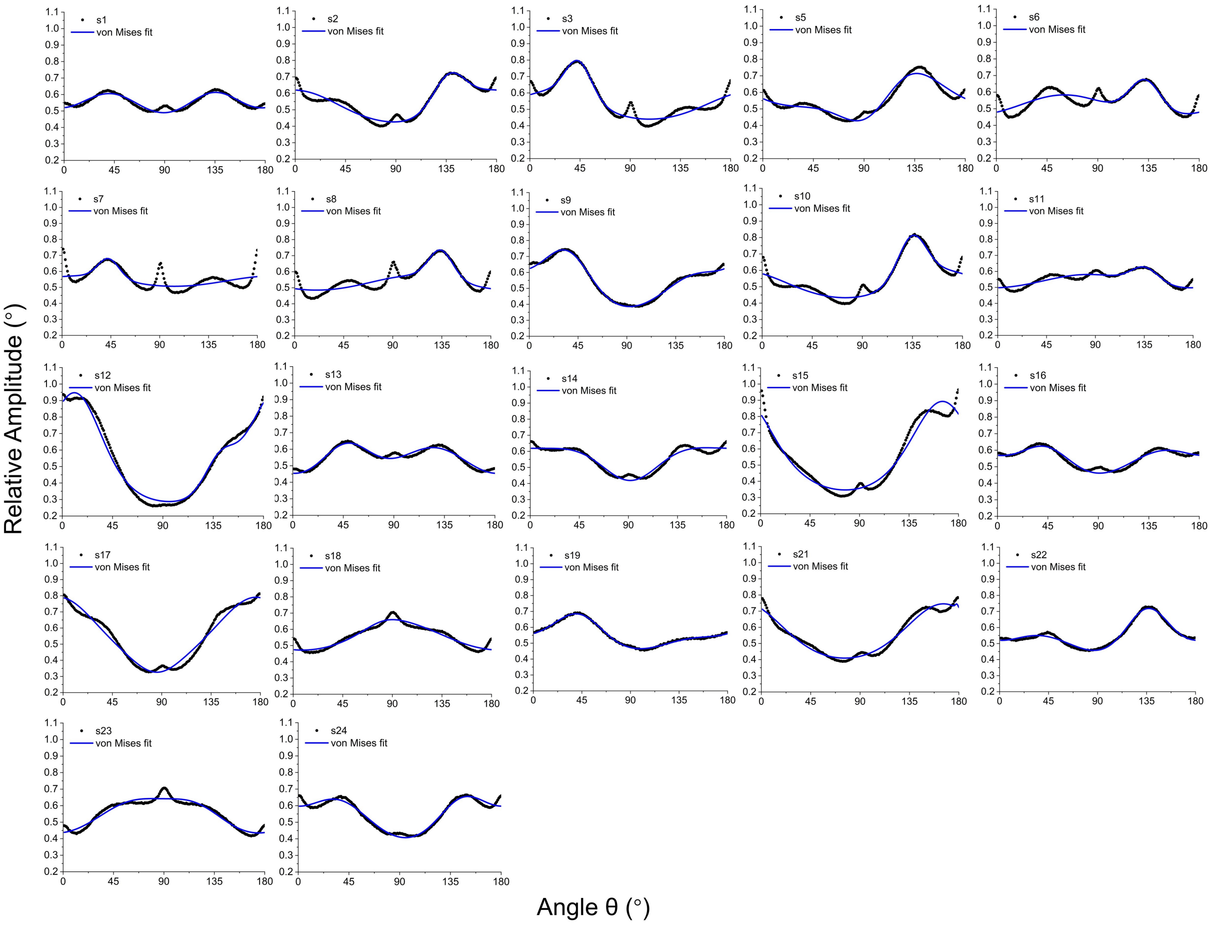

Fig. S7 Representative elastin fiber distributions of 24 samples fitted with von-Mises distribution.

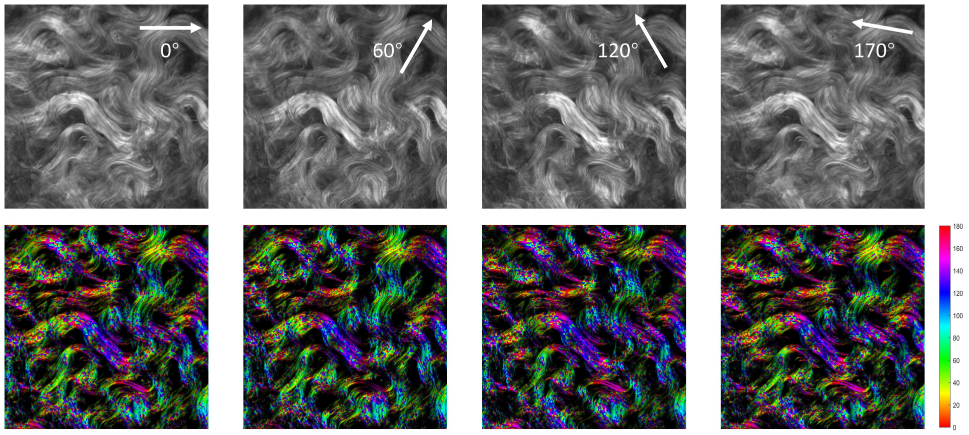

Fig. S8 Representation of SHG images and the orientation color map under diverse linear polarization angle light. White arrow indicates the polarization angle of the light. FOV: 200 × 200 μm^2. Note: the orientation analysis employed a sampling ratio parameter of 2.5 between the xy and z dimensions to match lateral resolutions of 0.2 μm/pixel and actual axial resolutions of 0.5 μm/pixel.

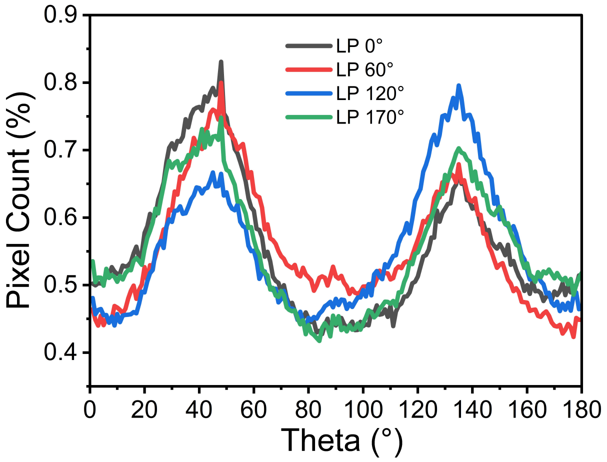

Fig. S9 Representative collagen fiber theta angle distribution under diverse linear polarization angle light.

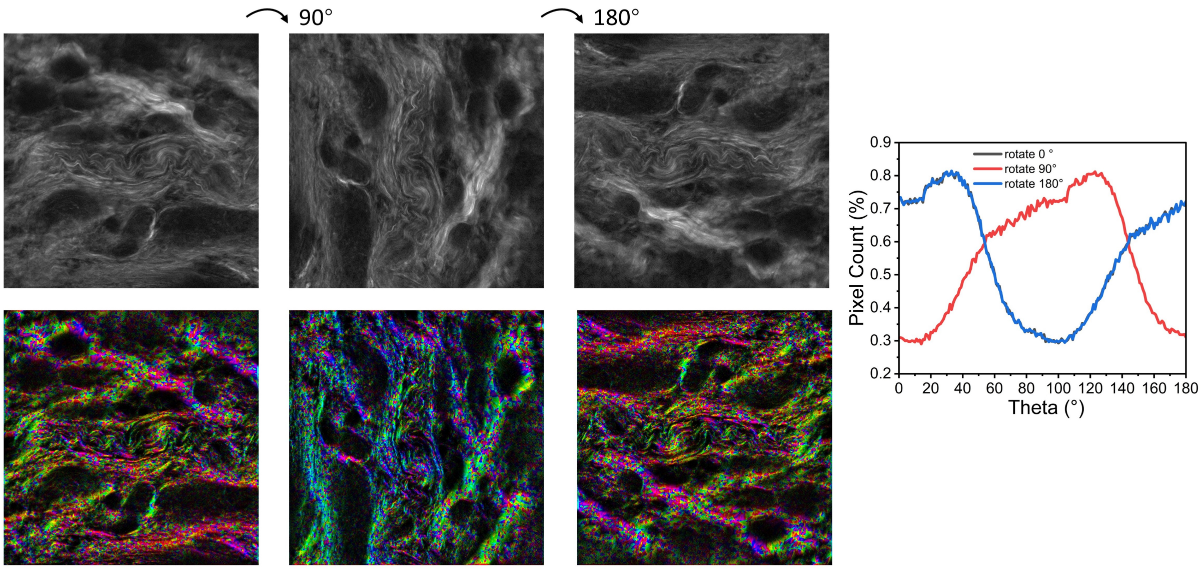

Fig. S10 SHG image rotation, the orientation color map and corresponding representative theta angle distribution. FOV: 200 × 200µm^2. Note: the orientation analysis employed a sampling ratio parameter of 2.5 between the xy and z dimensions to match lateral resolutions of 0.2 µm/pixel and actual axial resolutions of 0.5µm/pixel.

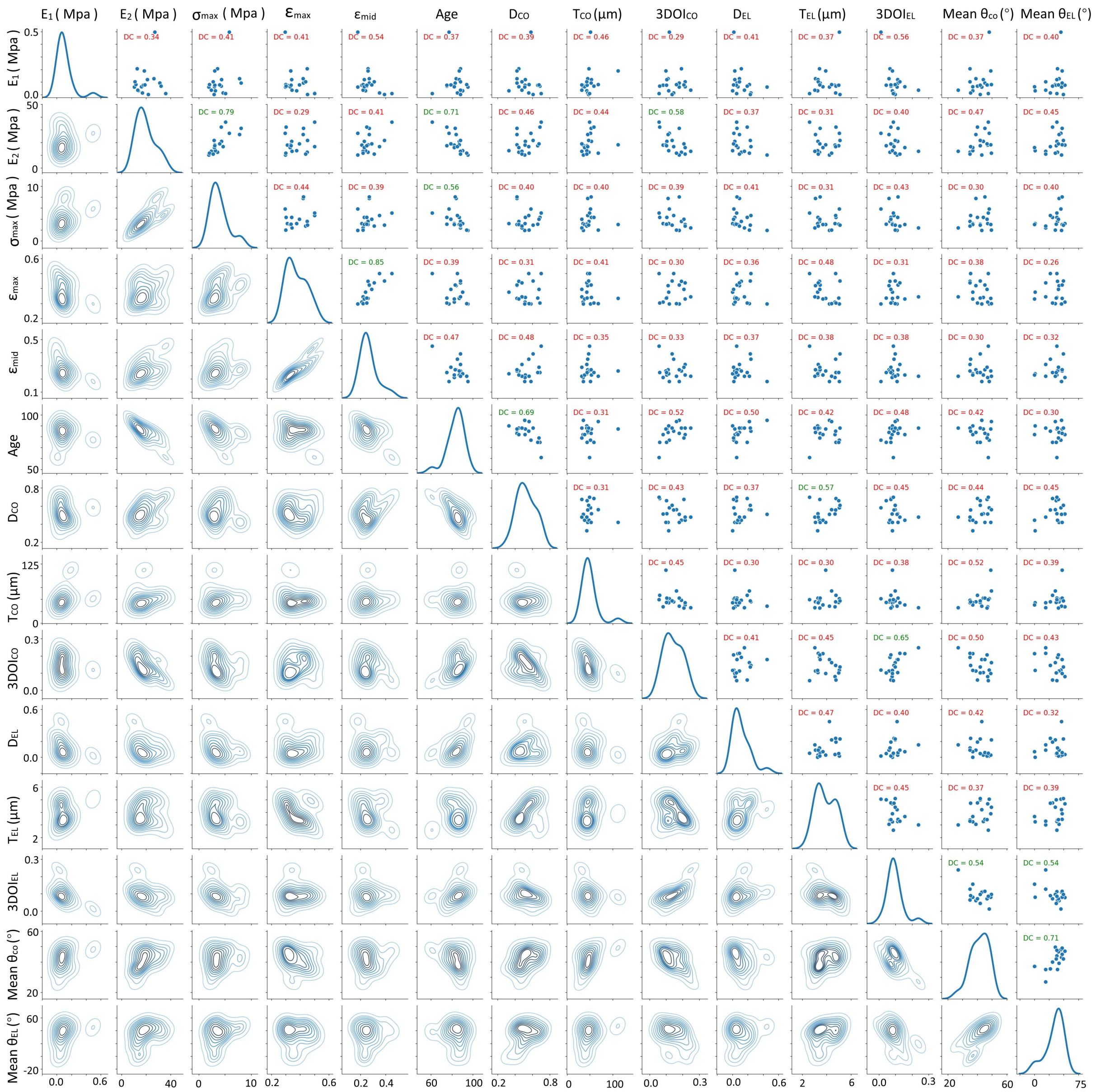

Fig. S11 The upper half of the diagram shows the scatter plots between the various variables and the distance correlation in green ($p < 0.05$) and red ($p > 0.05$). On the diagonal the kernel density estimations of the single variable distributions are shown. The lower half of the diagram shows the isolines of the kernel density estimations of the various bivariate distributions.

Supplementary Image data pre-processing

0.1 Self-supervised denoising

Since the two photon fluorescent (TPEF) channel not only collects elastin signals but also some of the collagen signals, the elastin signal is not pure. Now, because the TPEF signal is very weak this means that if we want to increase the TPEF signal, we also increase the collagen signal, which is unwanted. We therefore decided to increase the image quality of the elastin signal by removing background noise and small structures like lipids or flavins, since they are known to generate TPEF signal [1]. Usually a denoising deep learning model is trained in a supervised manner on a large number of pairs (x_i, y_i) of noisy inputs x_i and clean targets y_i. In practice we often only have access to multiple noisy instances of the same images thus making it impossible to formulate the denoising task as a supervised learning task. To overcome this issue, self-supervised training schemes such as NOISE2NOISE [2] and NOISE2VOID (N2V) [3] have been proposed. These two methods allow for denoising without reference to a clean ground truth. Both methods rely on the fact that by training on noisy input and target images only, the network will, on average, learn to restore the clean image. We chose the N2V denosing method since it allows for training on the noisy body of data itself and there are no free adjustable parameters, cf. non-local means denoising.

0.2 Training and validation

A 3D N2V network was trained on 25 z-stacks taken from the whole image dataset. The z-stacks were divided into patches of size $16 \times 64 \times 64$ pixels and a 75% -25% train-validation split was done. The N2V network was then trained for 100 epochs using the ADAM optimizer with a learning rate of 0.0004 and the network with the lowest validation loss was saved, see Figure S12. We used the JugLab N2V implementation (https://github.com/juglab/n2v) for TensorFlow 2.4.1. The training and inference were done on a NVIDIA GeForce GTX 1080 GPU with 8 GB of RAM on the BAZIS computational cluster of the Vrije Universiteit Amsterdam.

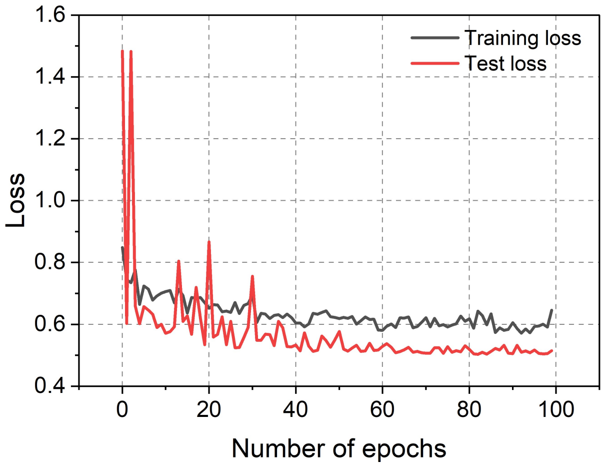

Fig. S12 The loss curves for both the training and validation data. The network with the lowest validation loss was saved.

There is no objective metric to evaluate the performance of the N2V training scheme. However, to better asses the performance of the N2V network, we additionally used non-local means (NLM) [4], a widely used powerful denoising method for the image set. NLM replaces the value of a pixel by an average of a selection of other pixels. We observed that NLM removes more noise, but also tends to blur the image. (The noise removed was estimated by a wavelet-based estimator of the noise standard deviation). This was observed across the whole dataset. We then further validated the denoising scheme qualitatively by careful visual inspection of the denoising results. Figure S13 shows an example of noisy and denoised elastin images at different depths. One can clearly see the elastin fiber structures are enhanced in the denoised image and that no artifacts are introduced.

The trained N2V network was then used to denoise the individual TPEF channels in the image z-stacks.

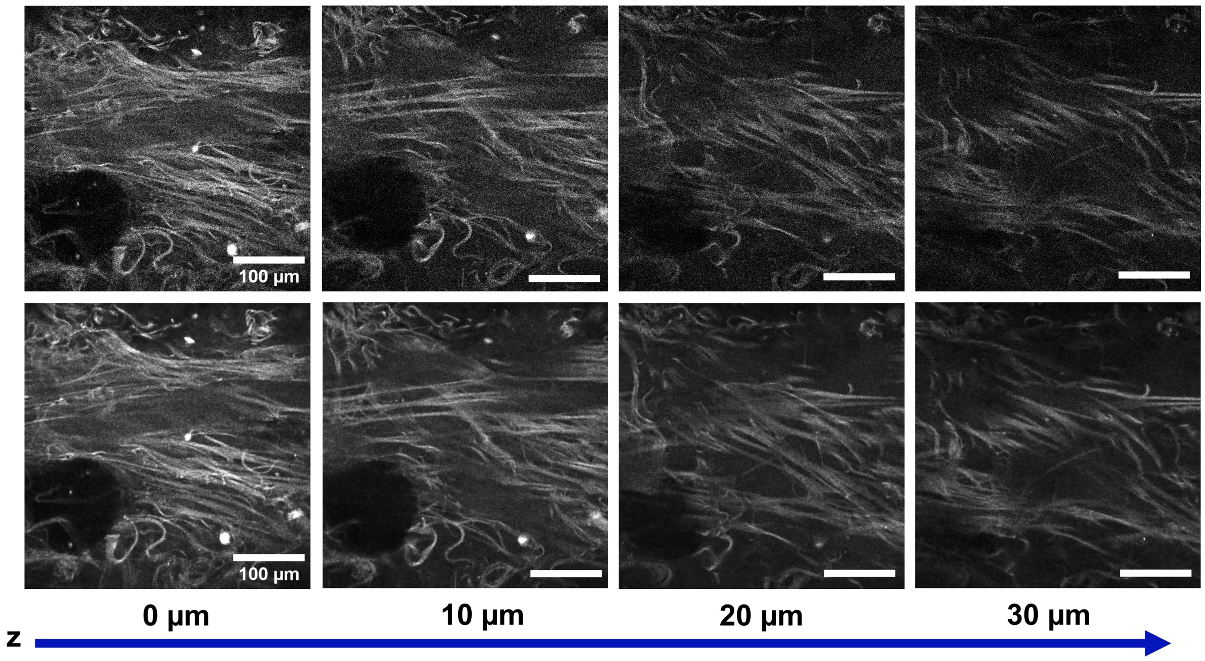

Fig. S13 Noisy and denoised elastin signals. The top row shows four 2D slices in different depth from a z-stack of S14. The bottom row shows the corresponding denoised images. The TPEF channel is shown in grey for visualization purposes. FOV: 400 × 400 µm^2

References

[1] Gina Fürtjes, David Reinecke, Niklas von Spreckelsen, Anna-Katharina Meißner, Daniel Rueß, Marco Timmer, Christian Freudiger, Adrian Ion-Margineanu, Florian Khalid, Konstantin Watrinet, et al. Intraoperative microscopic autofluorescence detection and characterization in brain tumors using stimulated raman histology and two-photon fluorescence. *Frontiers in Oncology*, 13:1146031, 2023.

[2] Jaakko Lehtinen, Jacob Munkberg, Jon Hasselgren, Samuli Laine, Tero Karras, Miika Aittala, and Timo Aila. Noise2noise: Learning image restoration without clean data. *arXiv preprint arXiv:1803.04189*, 2018.

[3] Alexander Krull, Tim-Oliver Buchholz, and Florian Jug. Noise2void - learning denoising from single noisy images. 2019.

[4] Antoni Buades, Bartomeu Coll, and Jean-Michel Morel. Non-Local Means Denoising. *Image Processing On Line*, 1:208–212, 2011. https://doi.org/10.5201/ipol.2011.bcm_nlm.
